# Supplementary material for: The impact of a named GP scheme on continuity of care and emergency hospital admission: a cohort study among older patients in England, 2012–2016
Source: BMJ Open. 2019 Sep 23;9(9):e029103. doi: 10.1136/bmjopen-2019-029103 (PMC6773345; doi:10.1136/bmjopen-2019-029103)
Supplement: Supplementary data [file bmjopen-2019-029103supp001.pdf]

Supplement

Supplementary Figure 1: Distribution of date when patients aged 75 and older were notified of named GP (n=12,526).

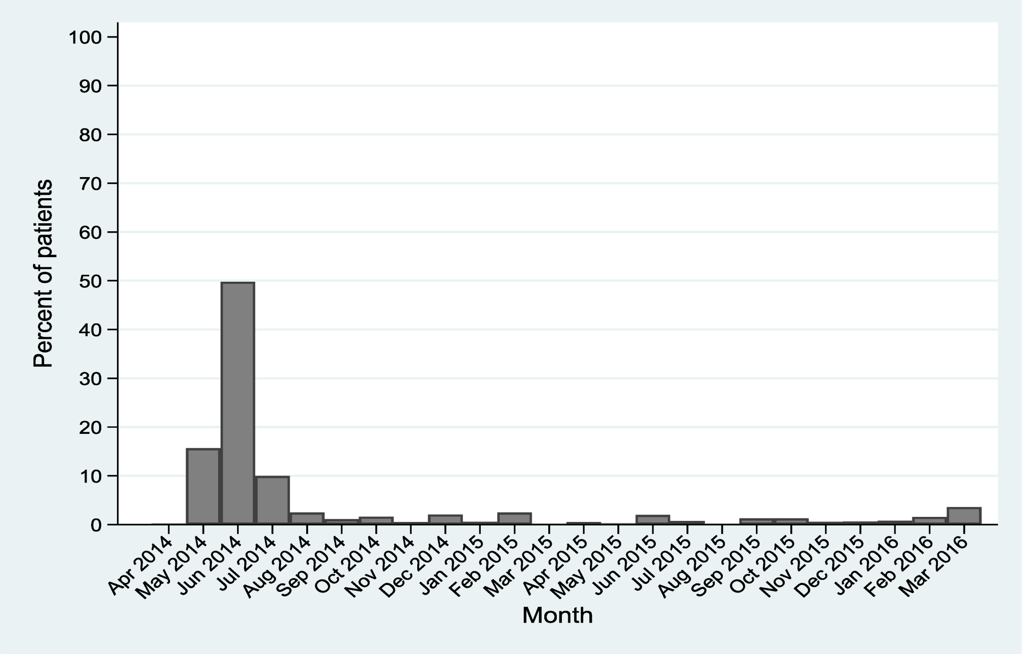

Supplementary Figure 2: Distribution of change in BB index-score between 2012-14 and 2014-16 for patients aged 65-70 (N=9,502) and 75 or older (N=8,409).

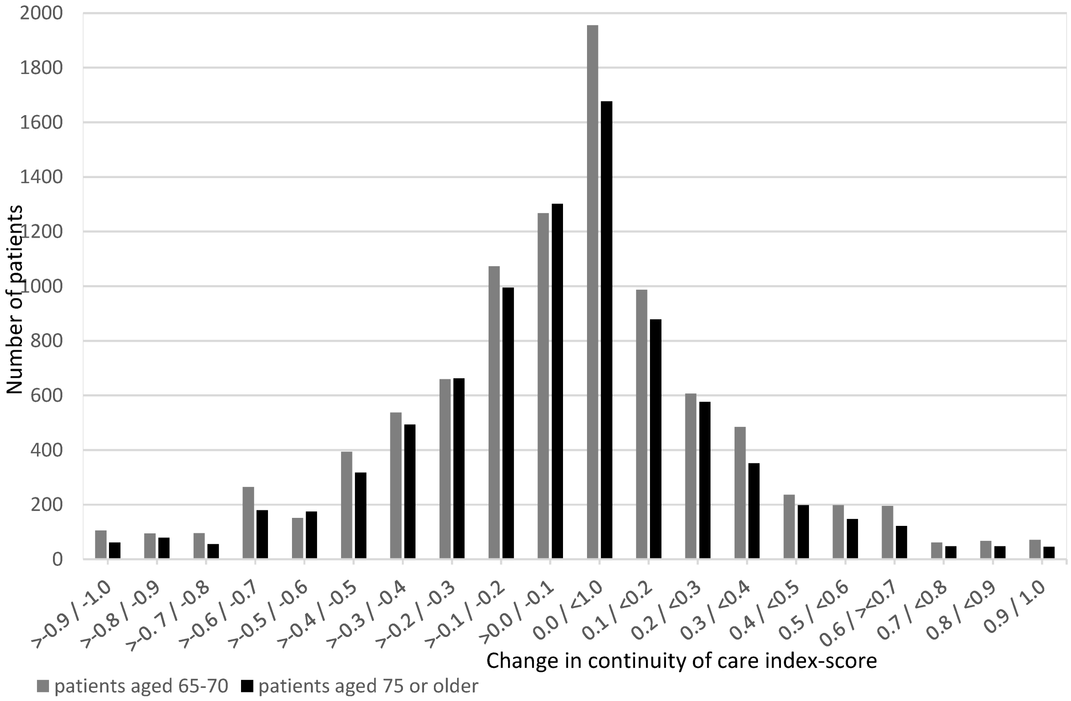

Supplementary Table 1: Cross -tabulation of continuity of care (using Bice & Boxerman index-scores) and number of GP consultations in England between 2012 and 2016 (38,500 observations).

CoC (BB index)	**Number of GP consultations (%)**				
	2-5	6-9	10-15	16+	
Lowest CoC	3,941 (40.8)	1,946 (20.2)	1,869 (19.4)	1,896 (19.6)	9,652 (100.0)
CoC Qr2	1,454(15.6)	2,417 (25.9)	2,456 (26.3)	3,017 (32.3)	9,344 (100.0)
CoC Qr3	2,036 (20.7)	2,361 (24.0)	2,566 (26.0)	2,889 (29.3)	9,852 (100.0)
Highest CoC	3,032 (31.4)	2,441 (25.3)	2,172 (22.5)	2,007 (20.8)	9,652 (100.0)
Total	10,463 (27.2)	9,165 (23.8)	9,063 (23.5)	9,809 (25.5)	38,500 (100.0)

CoC=continuity of care, Qr=quartile, BB=Bice & Boxerman

Supplementary Table 2: Estimates of B-coefficients from multi-level regression (normal response) model for the association between introduction of named-GP and continuity of care (Bice & Boxerman index-score), England 2012-2016 (38,500 observations), adjusted for standardised covariates.

	Coef.	95% CI	p-value
Constant	0.427	0.404; 0.449	<0.001
Period (ref.=pre)	-0.024	-0.030; -0.017	<0.001
Age (ref.= 75-)	0.017	0.009; 0.025	<0.001
Period * Age	-0.005	-0.014; 0.005	0.344
Female (ref.=male)	-0.007	-0.010; -0.004	<0.001
Number of chronic morbidities[1]	-0.006	-0.009; -0.003	<0.001
Deprivation level Qn2 (ref.= Qn1-Least deprived)	0.001	-0.003; 0.005	0.503
Deprivation level Qn3 (ref.= Qn1-Least deprived)	-0.001	-0.006; 0.003	0.490
Deprivation level Qn4 (ref.= Qn1-Least deprived)	-0.003	-0.007; 0.001	0.142
Deprivation level Qn5 (ref.= Qn1-Least deprived)	-0.003	-0.007; 0.000	0.085
Number of GP consultations Qr2 (ref.=Qr1-lowest number)	0.002	-0.001; 0.005	0.255
Number of GP consultations Qr3 (ref.=Qr1-lowest number)	-0.001	-0.004; 0.003	0.716
Number of GP consultations Qr4 (ref.=Qr1-lowest number)	-0.004	-0.007; -0.000	0.051
Number of GPs in practice Qr2 (ref.=Qr1-lowest number)	-0.037	-0.046; -0.028	<0.001
Number of GPs in practice Qr3 (ref.=Qr1-lowest number)	-0.050	-0.061; -0.040	<0.001
Number of GPs in practice Qr4 (ref.=Qr1-lowest number)	-0.069	-0.081; -0.057	<0.001
Cities and towns (ref.=urban conurbation)	0.023	-0.009; 0.055	0.152
Rural (ref.=urban conurbation)	-0.017	-0.051; 0.018	0.342
Practice-level variance (constant)	0.017	0.013; 0.021	
Patient-level variance (constant)	0.017	0.016; 0.018	
Period-level variance (constant)	0.053	0.052; 0.054	
intraclass correlation coefficient (ICC)	0.195		

[1] Diagnosed with one or more of the following seven chronic conditions: chronic renal disease, cancer, asthma, stroke, coronary heart disease, diabetes, or COPD. Ref.= reference; Qn=quintile; Qr=quartile.

Supplementary Table 3: Estimates of B-coefficients from multi-level regression (binomial logit) model for the association between introduction of named GP and risk of an emergency hospital admissions, England 2012-2016 (38,500 observations), adjusted for standardised covariates.

	Coef.	95% CI	p-value
Constant	-2.019	-2.088; -1.951	<0.001
Period (ref.=pre)	0.145	0.062; 0.229	0.001
Age (ref.= 75-)	0.466	0.381; 0.551	<0.001
Period * Age	0.175	0.064; 0.285	0.002
Female (ref.=male)	-0.028	-0.057; 0.002	0.066
Number of chronic morbidities[1]	0.465	0.438; 0.492	<0.001
Deprivation level Qn2 (ref.= Qn1-Least deprived)	0.304	-0.007; 0.068	0.109
Deprivation level Qn3 (ref.= Qn1-Least deprived)	0.054	0.016; 0.92	0.005
Deprivation level Qn4 (ref.= Qn1-Least deprived)	0.065	0.029; 0.102	<0.001
Deprivation level Qn5 (ref.= Qn1-Least deprived)	0.083	0.049; 0.117	<0.001
Number of GPs in practice Qr2 (ref.=Qr1-lowest number)	-0.008	-0.056; 0.040	0.750
Number of GPs in practice Qr3 (ref.=Qr1-lowest number)	-0.004	-0.054; 0.047	0.887
Number of GPs in practice Qr4 (ref.=Qr1-lowest number)	0.001	-0.053; 0.055	0.974
Cities and towns (ref.=urban conurbation)	-0.061	-0.119; -0.003	0.039
Rural (ref.=urban conurbation)	-0.022	-0.083; 0.039	0.482
Practice-level variance (constant)	0.020	0.008; 0.033	
Patient-level variance (constant)	0.548	0.465; 0.632	
Intraclass correlation coefficient (ICC)	0.035		

[1] Diagnosed with one or more of the following seven chronic conditions: chronic renal disease, cancer, asthma, stroke, coronary heart disease, diabetes, or COPD. Ref.= reference; Qn=quintile; Qr=quartile.

Note: the ratio of odds ratios for patients aged 75 and over was 1.19 compared with those under 75; this is calculated by taking e raised to the power of 0.175.

Supplementary Table 4: Estimates of B-coefficients from multi-level regression (Poisson) model for the association between introduction of named GP and the number of emergency hospital admissions, England 2012-2016 (38,500 observations), adjusted for standardised covariates.

	Coef.	95% CI	p-value
Constant	-1.868	-1.927; -1.808	<0.001
Period (ref.=pre)	0.164	0.098; 0.230	<0.001
Age (ref.= 75-)	0.452	0.380; 0.525	<0.001
Period * Age	0.134	0.051; 0.217	0.001
Female (ref.=male)	-0.024	-0.051; 0.003	0.087
Number of chronic morbidities[1]	0.428	0.403; 0.452	<0.001
Deprivation level Qn2 (ref.= Qn1-Least deprived)	0.036	0.002; 0.070	0.041
Deprivation level Qn3 (ref.= Qn1-Least deprived)	0.056	0.021; 0.91	0.002
Deprivation level Qn4 (ref.= Qn1-Least deprived)	0.070	0.037; 0.103	<0.001
Deprivation level Qn5 (ref.= Qn1-Least deprived)	0.086	0.055; 0.117	<0.001
Number of GPs in practice Qr2 (ref.=Qr1-lowest number)	0.004	-0.037; 0.045	0.845
Number of GPs in practice Qr3 (ref.=Qr1-lowest number)	0.014	-0.029; 0.058	0.525
Number of GPs in practice Qr4 (ref.=Qr1-lowest number)	0.004	-0.043; 0.050	0.887
Cities and towns (ref.=urban conurbation)	-0.058	-0.110; -0.006	0.028
Rural (ref.=urban conurbation)	-0.021	-0.076; 0.034	0.458
Practice-level variance (constant)	0.015	0.005; 0.025	
Patient-level variance (constant)	1.511	1.440; 1.582	
Intraclass correlation coefficient (ICC)	0.010		

[1] Diagnosed with one or more of the following seven chronic conditions: chronic renal disease, cancer, asthma, stroke, coronary heart disease, diabetes, or COPD. Ref.= reference; Qn=quintile; Qr=quartile.

Note: the ratio of rate ratios for patients aged 75 and over was 1.14 compared with those under 75; this is calculated by taking e raised to the power of 0.134.
