# Supplementary material for: The impact of a named GP scheme on continuity of care and emergency hospital admission: a cohort study among older patients in England, 2012–2016
Source: BMJ Open. 2019 Sep 23;9(9):e029103. doi: 10.1136/bmjopen-2019-029103 (PMC6773345; doi:10.1136/bmjopen-2019-029103)
Supplement: Author's manuscript [file bmjopen-2019-029103.draft_revisions.pdf]

BMJ Open

BMJ Open is committed to open peer review. As part of this commitment we make the peer review history of every article we publish publicly available.

When an article is published we post the peer reviewers' comments and the authors' responses online. We also post the versions of the paper that were used during peer review. These are the versions that the peer review comments apply to.

The versions of the paper that follow are the versions that were submitted during the peer review process. They are not the versions of record or the final published versions. They should not be cited or distributed as the published version of this manuscript.

BMJ Open is an open access journal and the full, final, typeset and author-corrected version of record of the manuscript is available on our site with no access controls, subscription charges or pay-per-view fees (http://bmjopen.bmj.com).

If you have any questions on BMJ Open's open peer review process please email info.bmjopen@bmj.com

BMJ Open

The impact of a named GP-scheme on continuity of care and emergency hospital admission. A cohort study among older patients in England, 2012-2016.

Journal:	*BMJ Open*
Manuscript ID	bmjopen-2019-029103
Article Type:	Research
Date Submitted by the Author:	14-Jan-2019
Complete List of Authors:	Tammes, Peter; University of Bristol, Bristol Medical School<br>Payne, Rupert; University of Bristol, Centre for Academic Primary Care<br>Salisbury, Chris; University of Bristol, Centre for Academic Primary Care<br>Chalder, Melanie; University of Bristol, Centre for Academic Primary Care<br>Purdy, Sarah; University of Bristol, Centre for Academic Primary Care<br>Morris, Richard; University of Bristol, School of Social & Community Medicine
Keywords:	Health policy < HEALTH SERVICES ADMINISTRATION & MANAGEMENT, Organisation of health services < HEALTH SERVICES ADMINISTRATION & MANAGEMENT, PRIMARY CARE, PUBLIC HEALTH, STATISTICS & RESEARCH METHODS

SCHOLARONE™
Manuscripts

The impact of a named GP-scheme on continuity of care and emergency hospital admission. A cohort study among older patients in England, 2012-2016.

Peter Tammes[1], Rupert Payne[1], Chris Salisbury[1], Melanie Chalder[1], Sarah Purdy[1] & Richard W Morris[1]

[1] University of Bristol, Bristol Medical School: Population Health Sciences, Centre for Academic Primary Care

Correspondence to Peter Tammes, Bristol Medical School: Population Health Sciences, University of Bristol, 39 Whatley Road, Bristol, BS8 2PS (e-mail: p.tammes@bristol.ac.uk) Phone: +44 (0) 117-928-7377; Fax: +44 (0) 117-928-7236

Key words: Health policy; organisation of health services; primary care; public health; statistics & research methods

Word count: 3769

Abstract

Objective:

To investigate whether the introduction of a named general practitioner (GP, family physician) improved patients' health care for patients aged 75 and over in the UK.

Setting

Random sample of 27,500 patients aged 65-84 in 2012 within 139 English practices from the Clinical Practice Research Datalink linked with Hospital Episode Statistics.

Design

Prospective cohort approach, measuring patients' GP consultations and emergency hospital admissions two years before and after the intervention. We created two age groups in order to compare patients who were or were not subject to the intervention: (i) patients aged 75 years and over and (ii) patients younger than 75 in both periods. Adjusted associations between the named-GP

For peer review only

scheme, continuity of care and emergency hospital admission were examined using multilevel modelling.

Intervention

National Health Service policy to introduce a named accountable GP for patients aged 75 and over in April 2014.

Main outcome measures

Study's outcome measures: a) continuity of care index-score, b) risk of emergency hospital admissions, c) number of emergency hospital admissions.

Results

The intervention was associated with a decrease in continuity index-scores of -0.024 (95% CI -0.030 to -0.017, p<0.001); there were no differences in the decrease between the two age groups (-0.005, 95% CI -0.014 to 0.005). The probability of an emergency hospital admission increased after the intervention (OR 1.156, 95% CI 1.064 to 1.257, p=0.001); this increase was bigger for patients 75 and over (relative OR 1.191, 95% CI 1.066 to 1.330, p=0.002). The average number of emergency hospital admissions increased after the intervention (RR 1.178, 95% CI 1.103 to 1.259, p<0.001); this increase was greater for patients 75 and over (relative RR 1.143, 95% CI 1.052 to 1.242, p=0.001).

Conclusion

The introduction of the named-GP scheme was not associated with improvements in either continuity of care or rates of unplanned hospitalisation.

Strengths and limitations of this study

- This study was the first to investigate the relationship between the introduction of a named GP-scheme and continuity of primary care and unplanned hospitalisation.
- This study took a 4-year observational period into account, namely 2-year periods before and after the implementation of the named-GP policy.
- This cohort study used individual electronic healthcare records data from the Clinical Practice Research Datalink (CPRD) linked with Hospital Episode Statistics.
- Our dataset did not make it possible to specify the named GP assigned to a patient.
- As all older patients included in the sample survived the 4-year observational period, this might reduce the generalisability of our findings.

For peer review only

Introduction

Nearly every country in the world is experiencing growth in the number and proportion of older persons.[1] It is projected that by 2019 one in eleven people in the United Kingdom (UK) will be aged 75 or older, increasing to one in seven by 2040[2]. This demographic trend is likely to increase the number and proportion of people with long-term disability and chronic or multiple health conditions. To meet the challenges of an ageing population and to better serve those living with complex health and care needs, in April 2014 the National Health Service (NHS) Employers and General Medical Services (GMS) agreed to introduce a named accountable general practitioner (GP) [family or primary care physician] for all patients aged 75 or more. The aim was to provide personalised, proactive care to keep older people healthy, independent and out of hospital.[3 4]

In the UK, patients are registered at one general (family) practice but might see different physicians within that practice. The introduction of a named GP-scheme was intended to facilitate older patients consulting the same doctor, thus improving continuity of primary care (i.e. seeing the same clinician over time). As there is some evidence that continuity of primary care is declining in the UK[5], the named-GP scheme could potentially reverse this trend.

A key objective of the introduction of the named-GP scheme was to avoid or decrease hospital admission. Previous systematic reviews based on international literature concluded that better clinician continuity of care reduces hospitalisation.[6 7] There is some evidence from the UK that patients who do not see the same GP over a period of time are at higher risk of emergency hospital admission and have more admissions than those who see the same or a small number of GPs.[8 9] The aim of the current study was to investigate whether the named-GP scheme improved older patients' continuity of primary care, and decreased older patients' risk of emergency hospital admission and the number of such admissions. The findings of this study might also be relevant for other (Western) countries as they face the same burdens from an ageing population on their health care systems.

Methods

<u>Study design and setting</u>

This study used data from the Clinical Practice Research Datalink (CPRD) which contains anonymised electronic healthcare records on 4.4 million patients (6.9% of the UK population) and is nationally representative in terms of age, sex and ethnicity.[10] We obtained a random sample of 27,500 patients in 139 English GP-practices [family practices] who were aged 65 to 84 in 2012, alive in 2016, registered with their GP-practice for at least one year prior to 2012, and not transferred out of the

For peer review only

GP-practice during the study's observation period. The CPRD was deterministically linked with Hospital Episode Statistics (HES) to identify emergency hospital admissions in the financial years April 2012 – March 2016. This allowed us to compare a patient's individual healthcare use two years before the introduction of the named GP-scheme (April 2012-March 2014) with their healthcare use two years after its introduction (April 2014-March 2016). To measure the impact of the introduction of a named-GP scheme appropriately, we excluded patients who became 75 years old during the study (n=5,703) and created two age groups for patients who were aged 75 years and over in both periods (n=9,682), or aged under 75 years in both periods (n=12,115) in order to compare groups who were or were not subject to the intervention. Furthermore, analyses were restricted to only those patients with at least two GP consultations allowing to calculate continuity of primary care scores in either the pre- or post-intervention periods, totalling 19,235 patients in the pre-intervention period and 19,265 patients in the post-intervention period.

Based on our previous work, we expect 13.5% to undergo an emergency admission over a single year, therefore over 2 years, we estimate 22% will experience an admission.[9] Comparing this with a subsequent 2-year period in which the rate is reduced by 10% (i.e. rate ratio of 0.9) to 19.8% by introduction of the named-GP scheme for patients aged 75 and over, we have over 99% power to detect this difference at the 5% significance level at the given sample size for the pre- and post-intervention periods. If the rate is reduced by 6% (instead of 10%) to 20.7%, we have 88% power to detect this difference.

Exposure

After the introduction of the named-GP scheme in April 2014, all patients aged 75 and over had to be notified by letter or during a consultation by 30 June 2014 of their named and accountable GP, or within 21 days after a patient became 75 or was newly registered if their practice was not already operating a personal list.[3] Practices were required to enter the Read Code "Informing patient of named accountable general practitioner" (code 67DJ) in the patient's health record. Based on the date of recording of this Read Code for all the 12,526 patients 75 and older in 2014 in the dataset, 65% were informed before 1 July 2014 and 75% before 1 August 2014. At the end of the observation period, i.e. 1 April 2016, 97% had been notified (see Supplementary Figure 1). We, however, did not exclude patients who were notified after 30 June 2014 for the purposes of our study as we did not know which practices already were operating a personal list. Furthermore, our analysis focused on the effects of the policy intervention as it was implemented; in effect, an intention-to-treat analysis.

Outcome measures

For peer review only - http://bmjopen.bmj.com/site/about/guidelines.xhtml

Three outcome measures were assessed, the first being change in continuity of primary care. We used a combination of CPRD staff codes to identify GP staff (senior partners, partners, salaried doctors, locum doctors, and GP registrars) within the practice and dates of consultations to identify whether these occurred during the pre-intervention or post-intervention period. Consultations included clinic and surgery consultations, home visits, out-of-hours' visits, telephone consultations, and third-party consultations. Following Hobbs et al. we did not restrict according to consultation duration.[11] Where a patient had more than one contact per day, we used information about staffing relating to the first consultation only, to avoid potential concerns about duplication of consultations. This information allows the quantification of continuity of care over 2-year periods pre-intervention and post-intervention. We calculated continuity of care using the Bice & Boxerman (BB) index[12], which ranges between 0 (complete absence of continuity) and 1 (perfect continuity of care), as this has been recommended for use in primary care research[13]. BB index-scores can be calculated for patients who consulted a GP more than once.

The second outcome assessed was change in probability of an emergency hospital admission after the second GP-consultation in both the pre- and post-intervention periods. We made no distinction between admissions by specific routes (e.g. patient self-presentation to the emergency department, GP referral to a hospital speciality). In the pre-intervention and the post-intervention periods, respectively, 2,966 (15.4%) and 3,745 (19.4%) patients had one or more emergency admissions.

The third outcome was the number of emergency hospital admissions after the second GP-consultation in both the pre- and post-intervention periods, categorised as zero, one, two, and three or more. In the pre-intervention period 16,269 (84.6%), 2,070 (10.7%), 557 (2.9%), and 339 (1.76%) experienced respectively no, one, two, or three or more emergency hospital admissions. In the post-intervention period 15,520 (80.6%), 2,368 (12.3%), 750 (3.9%), and 627 (3.3%) experienced respectively no, one, two, or three or more emergency hospital admissions.

For peer review only

Covariates

Our choice of covariates was guided by the QAdmission score[14], previously developed using data from a similar routine general practitioner database to predict hospital admissions. Complete data were available on all patients in the sample with regard to age, gender, number of GP consultations, area-based socioeconomic deprivation in quintiles, location (conurbation, urban, rural), and the following morbidities measured between April 2010 and March 2014 for the pre-intervention period and measured between April 2012 and March 2016 for the post-intervention period. These included diagnoses made in the 2 years prior to the pre- and post-intervention periods, using published clinical code lists as collected in the Manchester Clinical Codes repository[15]: epilepsy[16], chronic

kidney disease[17], cancer[18], asthma[17], stroke[19], coronary heart disease[19], diabetes[19], COPD[16], depression[20], and schizophrenia[20]. Furthermore, we took into account clustering at the practice level as practice factors might facilitate or reduce continuity of care[21] and estimated the number of GPs in a practice using patient's GP consultations and staff role information for each general practice. Descriptive statistics are provided in Table 1.

To adjust for continuity of care (BB index-score) for the second outcome, we determined the continuity of care until an emergency hospital admission or to the end of each period (whichever came first) when not having experienced such an emergency admission, excluding from the analysis patients who experienced an emergency admission before their first or second GP consultation (as these patients would not have a continuity score). This resulted in a reduction in the of number of observations from 38,500 to 37,207.

Table 1 here

Statistical methods

A patient's BB index and a patient's emergency hospital admission were measured for both the pre-intervention and the post-intervention periods. To account for repeated measurements by time, by patient and by practice, this study used multilevel modelling. A normal response regression model was used to associate the named-GP scheme with continuity of care (BB index-score), a binomial logit regression model to associate the named-GP scheme with risk of emergency hospital admissions, and a Poisson regression model to associate the named-GP scheme with the number of emergency hospital admissions.

To represent whether the effect of the intervention operated differently for patients aged over 75 (exposed) from those aged under 75 (unexposed), we included the age × period interaction. This could be interpreted as difference in change of the BB index score, the relative odds ratio of emergency hospital admission or the relative rate ratio of number of emergency hospital admissions.

Results

Outcome: continuity of primary care

The distribution of the BB index varied widely, with similarity between the pre and post-intervention distributions (Figure 1). Respectively, 1,365 (7.1%) and 2,523 (13.1%) patients never or always saw

For peer review only - http://bmjopen.bmj.com/site/about/guidelines.xhtml

the same GP in the pre-intervention period: equivalent numbers were 1,376 (7.1%) and 2,086 (10.8%) in the post-intervention period.

Figure 1 here

The BB index-score decreased over time by 0.028 (from 0.428 in the pre-intervention period to 0.399 in the post-intervention). The BB index-score for patients aged 75 and over decreased from 0.434 pre-intervention to 0.403 post-intervention (a mean decrease of 0.031). This decrease was slightly bigger than for patients younger than 75, from 0.422 pre-intervention to 0.397 post-intervention (a drop of 0.025). An unadjusted multilevel (normal response) model for continuity of care (BB index-score) showed there was no evidence that this decrease in continuity of care following the intervention differed between the two age groups (Table 2, time-age interaction -0.006 (95% CI -0.015; 0.004)). As patients in the lowest or highest continuity of care quartiles consulted a GP less often than those in the middle two quartiles (see Supplementary Table 1), we included number of consultations in the analysis as one of the co-variates together with other factors such as gender, number of chronic conditions, socioeconomic deprivation, number of GPs in practice, and rurality. This adjusted model still showed no significant difference in the decrease of continuity of care over time between the two age groups (Table 2, time-age interaction -0.005 (95%CI -0.014; 0.005)). Continuity of care declined in both the unexposed and exposed groups and there was no evidence of the decline being stronger in one of the groups.

Table 2 here

We also calculated for each practice the average practice-level continuity of care score over 2012-2014, having divided practices into tertiles: low, middle, high continuity of care. This allowed us to determine whether patients in practices with different levels of continuity of care show differing trends in continuity of care post-intervention. The result of an interaction between period, age, and practice-level continuity of care is illustrated in Figure 2. The continuity of care of patients in a practice with generally low continuity of care dropped less between pre- and post-introduction of the named-GP scheme for both patients younger and older than 75 compared to practices with generally middle and high practice-level continuity of care.

Figure 2 here

Outcome: risk of emergency hospital admission

The probability of an emergency hospital admission for patients aged 75 and over showed an absolute increase of 6.3%-points over time (from 19.9% pre-intervention to 26.2% post-intervention). There was evidence from the unadjusted model that the relative increase in odds of

For peer review only - http://bmjopen.bmj.com/site/about/guidelines.xhtml

admission was 17.9% (95%CI 5.9%-31.4%) greater in those aged over 75 years than those aged under 75 years after the introduction of the named-GP scheme (Table 3). This relative difference between age groups persisted (19.1%, 95%CI 6.6%-33.0%) after adjustment for other factors such as gender, number of chronic conditions, socioeconomic deprivation, number of GPs in practice, and rurality. The relative difference between age groups was marginally greater following additional adjustment for continuity of care (BB index-score estimated until the event date) and number of GP consultations (22.8%, 95%CI 8.6%-38.8%).

Table 3 here

<u>Outcome: number of emergency hospital admissions</u>

The mean number of emergency hospital admissions for patients aged 75 and over showed an absolute increase of 0.154 over time (from 0.313 pre-intervention to 0.467 post-intervention). There was evidence from the unadjusted model that the relative increase in mean number of emergency hospital admissions after the introduction of the named-GP scheme was 14.6% (95%CI 5.5%-24.5%) greater in those aged over 75 years than those aged under 75 years (Table 4, Unadjusted model). This relative difference between age groups persisted (14.3%, 95%CI 5.2%-24.2%) after adjustment for other factors such as gender, number of chronic conditions, socioeconomic deprivation, number of GPs in practice, and rurality (Table 4, Adjusted model 1).

To adjust for continuity of care (BB index-score) and for number of GP consultations we determined the continuity of care at the end of each period for all patients included in the analysis, in contrast to the other outcome where continuity of care was estimated only until the event date. In this model the relative difference between the two age groups was slightly lower than in the unadjusted model (12%, 95%CI 3.1-21.5%) (Table 4, Adjusted model 2).

Table 4 here

Discussion

<u>Principal findings</u>

Continuity of care decreased between the pre- and post-intervention periods and this decrease was similar for patients aged between 65 and 74 (who were not eligible for the named-GP scheme over the period of study) and patients aged 75 and over (who were eligible). Over time, continuity of care for patients aged 75 years or over declined less in practices which had lowest continuity of care at baseline. The probability of an emergency hospital admission increased between the pre- and post-

For peer review only - http://bmjopen.bmj.com/site/about/guidelines.xhtml

intervention periods and this increase was greater for patients 75 and over. The average number of emergency hospital admissions also increased, and this increase was greater for patients aged 75 and over. In general, the introduction of a named-GP scheme was not associated with improvements in either continuity of care or rates of emergency hospital admissions.

Emergency hospital admission showed a stronger increase among patients aged 75 and over, contrary to what we expected, but we don't think that this is associated to the measured decrease in continuity of care as patients 75 and over and those younger than 75 experienced a similar drop in continuity. It is unclear whether the increase is due to the named-GP system mediated through some other mechanism than continuity of care, or whether it's due to other factors not captured by our study.

Strengths and weaknesses of the study

This study used longitudinal individual-level data from older patients in the CPRD to assess continuity of care and its relationship with the incidence of unplanned hospital admission before and after the introduction of a named GP-scheme, by comparing patients assigned a named GP with slightly younger patients not assigned a named GP. This allowed us to determine and compare continuity of care and unplanned hospital admission over time and between the affected and unaffected group. The observation period was a 2-year period before and after the introduction of a named GP-scheme, allowing us to calculate robust continuity of care scores for each period using the BB index-scores. The dataset allowed us also to control for practice-level, clinical and demographic co-variates.

The study had some limitations. Firstly, all the patients included in the sample survived the 4-year observational period. This may indicate that we had a particularly 'healthy' group of older patients and might, therefore, reduce the generalisability of the study's findings. Our dataset did not make it possible to specify the named GP assigned to a patient, therefore we could not use other measures of continuity of care such as the provider identification index.[22]

Comparison with other studies

Lloyd and Steventon published a protocol for a regression discontinuity study to investigate the effect of the introduction of the named GP-scheme on the number of GP contacts per patients, the number of GP referrals to specialists, and the number of common diagnostic tests.[23] Following up on their protocol, Barker, Lloyd and Steventon did not find any associations with their outcomes of interest measured over 9 months following assignment to a named accountable GP and attributed this to their short period under study.[24] The present study took a longer observational period into

For peer review only - http://bmjopen.bmj.com/site/about/guidelines.xhtml

account, namely 2-year periods before and after the implementation, resulting in more robust findings. Whereas study of Barker, Lloyd and Steventon focused on number of GP contacts, our study explored the possible effect on continuity of care, since one of the mechanisms by which assigning a named GP to a patient could have an impact might be by increasing continuity. Furthermore, Lloyd and Steventon's outcome measures reflect only primary care service use. As a key objective of the introduction of a named GP-scheme was to avoid or decrease hospital admission, this study also calculated associations between the introduction of a named GP and risk and number of emergency hospital admissions.

Using aggregated practice-level data from the GP-patient survey, Levene et al. showed that the proportion of patients seeing their preferred GP dropped between 2012 and 2017, especially in practices with higher percentages of those aged 75 and older.[25] Based on this result they questioned the effectiveness of the named GP-policy for older patients. Our study showed a decrease as well in continuity of care, measured by BB index-scores. However, as our study used individual-data, avoiding the ecological fallacy, we showed that the decrease in continuity of care was similar for those aged between 65-74 and for those aged 75 and older. Possibly because most patients were already listed at a GP list and introducing a named-GP policy for older patients might not have changed much to their situation of being allocated to a GP. We were also able to determine that continuity of care of patients in a practice providing on average low continuity of care dropped less compared to patients in practices providing on average high continuity of care, which may be an example of regression to the mean.

Meaning of the study: possible explanations and implications for clinicians and policymakers

The named GP-scheme for older patients was introduced by the NHS, with each individual general practice having to assign doctors to older patients on their list. The General Medical Services Contract did not advise practices to consult patients about their preferred GP as part of this assignment process, nor did it guarantee that patients would see the same clinician at each consultation. However, where patients expressed a preference as to which GP they have been assigned, the practice had to make reasonable effort to accommodate these requests.[3] In most general practices in the UK patients were already nominally allocated to a particular GP within a practice on the practice computer system, because until 2004 patients were registered with an individual GP rather than a practice. However, patients may not have been aware of this, and the GP named on the computer system may have had little or no significance for patient care.[26] The main change introduced with the named GP policy was informing patients of the GP who was accountable

For peer review only - http://bmjopen.bmj.com/site/about/guidelines.xhtml

for their care. This did not necessarily reflect which GP the patient had seen most often or take into account whether the patient had a preferred GP.

Allocating a GP does not imply that patients are able to see or speak to that GP whenever they require advice or care since this depends on GP workload, practice opening hours, salaried and part-time working contracts.[27] The importance of continuity of care in the patient-doctor relationship is much more complex than the simple allocation of a named doctor. Other factors that may be important, particularly in the context of reducing future emergency admissions, are the education of patients over a period of time, and knowledge of a patient's usual health status.[28] These are reflections of the depth of the relationship between the patient and doctor – which will not automatically be improved by the allocation of a named doctor to a particular patient.[29]

Unanswered questions and future research

Future research might focus on differences between practices concerning the implementation of the named GP scheme. As this research showed a difference between patients in general practices with on average low versus high continuity of care, a number of other differences could impact implementation, such as practice size and proportion of part-time GPs. Our study focused on continuity of care and unplanned hospital admission, future research using a 2-year or even longer observational period might focus on other healthcare use such as number of GP referrals or diagnostic tests[24], drug prescription and medication adherence. A complication, however, might be the introduction of a named-GP scheme for all patients in April 2015 which should have been implement in all practices before April 2016.[30] Though, the named GP for patients younger than 75 has largely a role of oversight for a patient's health in contrast to the named GP for patients 75 and over who should actively provide personalised care.

This study does not investigate the views and experiences of patients or practice staff. Evidence suggests that older patients value continuity of care[27], but qualitative research or surveys could explore whether they identified any change in care after the introduction of the named GP scheme. We also do not know whether the scheme led to any meaningful changes in how practices offered care to patients and or in the extent to which individual GPs felt accountable for particular patients. Qualitative research in practices could usefully explore this issue in order to improve implementation of a named GP-scheme.

For peer review only - http://bmjopen.bmj.com/site/about/guidelines.xhtml

Data Availability

Our data were obtained through licensing agreements (Protocol 17_140R) with the Clinical Practice Research Datalink (CPRD). The CPRD is the new English National Health Service (NHS) observational data and interventional research service, jointly funded by the NHS National Institute for Health Research (NIHR) and the Medicines and Healthcare products Regulatory Agency (MHRA). All access and use of data via the CPRD are carefully controlled under UK and European law and the rules and regulations operating in the NHS. In accordance with the conditions of the CPRD license, data abstracts from the CPRD could not be deposited in a public data depository. Data are available to other researchers upon request from the CPRD (http://www.cprd.com/contact/), and approval by the CPRD Independent Scientific Committee (ISAC, http://www.cprd.com/ISAC/).

Competing interests

None declared

Authors' contribution

PT and RWM designed the study. PT managed and performed the analysis. CS, MC, RP, and SP contributed to the methodological approach and also added significant input to the results and discussion. All authors contributed to the interpretation of the findings. PT was the lead investigator for the overall project.

Patient and Public Involvement in Research

At the start of the study, PT presented an outline of the project to the Elizabeth Blackwell Institute (EBI) Public Advisory Group based at the University of Bristol. Feedback from this meeting was used by MC to plan a range of engagement and involvement activities with older people and, where appropriate, their carers and family members. All activities took place in community settings, focusing on the concept of the policy initiative, the planned research design and the study results. Information from both the EBI Public Advisory Group and the discussions with patients and members of the public guided the preparation of data for analysis and informed the interpretation of the results. A plain English summary of the study findings has been prepared for dissemination alongside the scientific and academic outputs. A series of appropriately targeted publications for lay audiences has also been planned.

Data sharing

The data controller of the data analysed is the Clinical Practice Research Datalink. Patient level data is available subject to their information governance requirements.

For peer review only - http://bmjopen.bmj.com/site/about/guidelines.xhtml

Ethical approval

This study was approved by the CPRD ISAC committee.

Transparency declaration

The lead author affirms that this manuscript is an honest, accurate, and transparent account of the study being reported; that no important aspects of the study have been omitted; and that any discrepancies from the study as planned (and, if relevant, registered) have been explained.

Statements

This work was funded by the National Institute for Health Research School of Primary Care Research (NIHR SPCR) grant funded round 13, PI PT project number 337. The views expressed are those of the authors and not necessarily those of the NIHR, NHS or Department of Health.

This study is based in part on data from the Clinical Practice Research Datalink obtained under licence from the UK Medicines and Healthcare products Regulatory Agency. The data is provided by patients and collected by the NHS as part of their care and support. The interpretation and conclusions contained in this study are those of the author/s alone.

Copyright © 2017, re-used with the permission of The Health & Social Care Information Centre. All rights reserved.

References

1. United Nations, Department of Economic and Social Affairs, Population Division. World Population Ageing 2017 (ST/ESA/SER.A/408). New York, 2017.
2. Government Office for Science. Future of an Ageing Population. Foresight Report. London: Government Office for Science, 2016.
3. General Medical Services Contract 2014/15 Guidance and Audit Requirements. NHS England Gateway reference: British Medical Association, 2014.
4. Lea W. Transforming primary care. Safe, proactive, personalised care for those who need it most. London: Department of Health, 2014.
5. Baker M, Jeffers H. Continuity of care in modern day general practice. London: royal College of General Practitioners, 2016.
6. Van Walraven C, Oake N, Jennings A, et al. The association between continuity of care and outcomes: a systematic and critical review. *Journal of evaluation in clinical practice* 2010;16(5):947-56.
7. Huntley A, Lasserson D, Wye L, et al. Which features of primary care affect unscheduled secondary care use? A systematic review. *BMJ open* 2014;4(5):e004746.
8. Barker I, Steventon A, Deeny S. Continuity of care in general practice is associated with fewer hospital admissions for ambulatory care sensitive conditions: a cross-sectional study of routinely collected, person-level data. *BMJ* 2017

For peer review only - http://bmjopen.bmj.com/site/about/guidelines.xhtml

9. Tammes P, Purdy S, Salisbury C, et al. Continuity of primary care and emergency hospital admissions among older patients in England. *The Annals of Family Medicine* 2017;15(6):515-22.
10. Herrett E, Gallagher AM, Bhaskaran K, et al. Data Resource Profile: Clinical Practice Research Datalink (CPRD). *International journal of epidemiology* 2015;44(3):827-36.
11. Hobbs FR, Bankhead C, Mukhtar T, et al. Clinical workload in UK primary care: a retrospective analysis of 100 million consultations in England, 2007–14. *The Lancet* 2016;387(10035):2323-30.
12. Bice TW, Boxerman SB. A quantitative measure of continuity of care. *Medical care* 1977;15(4):347-49.
13. Salisbury C, Sampson F, Ridd M, et al. How should continuity of care in primary health care be assessed? *British Journal of General Practice* 2009;59(561):e134-e41.
14. Hippisley-Cox J, Coupland C. Predicting risk of emergency admission to hospital using primary care data: derivation and validation of QAdmissions score. *BMJ open* 2013;3(8):e003482.
15. Springate DA, Kontopantelis E, Ashcroft DM, et al. ClinicalCodes: an online clinical codes repository to improve the validity and reproducibility of research using electronic medical records. *PloS one* 2014;9(6):e99825.
16. Reilly S, Olier I, Planner C, et al. Inequalities in physical comorbidity: a longitudinal comparative cohort study of people with severe mental illness in the UK. *BMJ open* 2015;5(12):e009010.
17. Doran T, Kontopantelis E, Valderas JM, et al. Effect of financial incentives on incentivised and non-incentivised clinical activities: longitudinal analysis of data from the UK Quality and Outcomes Framework. *Bmj* 2011;342:d3590.
18. Reeves D, Springate DA, Ashcroft DM, et al. Can analyses of electronic patient records be independently and externally validated? The effect of statins on the mortality of patients with ischaemic heart disease: a cohort study with nested case–control analysis. *BMJ open* 2014;4(4):e004952.
19. Kontopantelis E, Springate D, Reeves D, et al. Withdrawing performance indicators: retrospective analysis of general practice performance under UK Quality and Outcomes Framework. *Bmj* 2014;348:g330.
20. Carr MJ, Ashcroft DM, Kontopantelis E, et al. Clinical management following self-harm in a UK-wide primary care cohort. *Journal of affective disorders* 2016;197:182-88.
21. Kristjansson E, Hogg W, Dahrouge S, et al. Predictors of relational continuity in primary care: patient, provider and practice factors. *BMC family practice* 2013;14(1):72.
22. Starfield BH, Simborg DW, Horn SD, et al. Continuity and coordination in primary care: their achievement and utility. *Medical care* 1976;14(7):625-36.
23. Lloyd T, Steventon A. Effect of Named, Accountable GPs on Continuity of Care: Protocol for a Regression Discontinuity Study of a National Policy Change. *International Journal of Integrated Care* 2016;16(1)
24. Barker I, Lloyd T, Steventon A. Effect of a national requirement to introduce named accountable general practitioners for patients aged 75 or older in England: regression discontinuity analysis of general practice utilisation and continuity of care. *BMJ Open* 2016;6(9):e011422.
25. Levene LS, Baker R, Walker N, et al. Predicting declines in perceived relationship continuity using practice deprivation scores: a longitudinal study in primary care. 2018:bjgp18X696209.
26. Goodwin N, Dixon A, Poole T, et al. Improving the quality of care in general practice: Report of an independent inquiry commissioned by The King's Fund. 2011
27. Palmer W, Hemmings N, Rosen R, et al. Improving access and continuity in general practice: practical and policy lessons. Research summary. London: Nuffield Trust, 2018.
28. Simmonds RL, Shaw A, Purdy S. Factors influencing professional decision making on unplanned hospital admission: a qualitative study. *Br J Gen Pract* 2012;62(604):e750-e56.

For peer review only - http://bmjopen.bmj.com/site/about/guidelines.xhtml

29. Merriel SWD, Salisbury C, Metcalfe C, et al. Depth of the patient–doctor relationship and content of general practice consultations: cross-sectional study. *Br J Gen Pract* 2015;65(637):e545-e51.
30. British Medical Association, NHS Employers, NHS England. Technical requirements for 2015/16 gms contract changes, 2015.

For peer review only

For peer review only - http://bmjopen.bmj.com/site/about/guidelines.xhtml

Table 1: Descriptive statistics

	Pre-intervention (19,235)	Post-intervention (19,265)
	N (Pct.)	Pct.
Aged <75	10,404 (54.1)	10,368 (53.8)
Aged > 74	8,831 (45.9)	8,897 (46.2)
Male	8,699 (45.2)	8,698 (45.2)
Female	10.536 (54.8)	10,567 (54.8)
Imd2015 least deprived, quintile 1	5,294 (27.5)	5,340 (27.7)
Imd2015 quintile 2	4,395 (22.9)	4,421 (23.0)
Imd2015 quintile 3	4,266 (22.6)	4,238 (22.0)
Imd2015 quintile 4	3,195 (16.6)	3,194 (16.6)
Imd2015 most deprived, quintile 5	2,084 (10.8)	2,071 (10.7)
Least number of GP consultations (2-5)	5,333 (27.7)	5,130 (26.6)
Q2_N_GPconsults (6-9)	4,697 (24.4)	4,468 (23.2)
Q3_N_GPconsults (10-15)	4,545 (23.6)	4,518 (23.5)
Q4_N_GPconsults (16+)	4,660 (24.2)	5,149 (26.7)
Least number of GPs in practice (pre: <9) (post: <8)	2,977 (15.5)	2,764 (14.4)
Q2_no of GPs (pre: 9-14) (post: 8-13)	4,715 (24.5)	4,555 (23.6)
Q3_no of GPs (pre: 15-21) (post: 14-22)	5,032 (26.2)	5,996 (31.1)
Q4_no of GPs (pre: >21) (post >22)	6,511 (33.9)	5,950 (30.9)
Urban conurbation	6,145 (32.0)	6,180 (32.1)
Cities and towns	10,207 (53.1)	10,290 (53.4)
Rural	2,883 (15.0)	2,795 (14.5)
No Emergency hospital admission	16,269 (84.6)	15,520 (80.6))
1 Emergency hospital admission	2,070 (10.8)	2,368 (12.3)
2 Emergency hospital admissions	557 (2.9)	750 (3.9)
>2 Emergency hospital admissions	339 (1.7)	627 (3.2)
	Median (IQ)	Median (IQ)
Total number of morbidities[1] (0-6)	1 (0-1)	1 (0-1)
Continuity of care (BB index-score) patient-level (0-1)	0.344 (0.184-0.622)	0.333 (0.167-0.574)
Continuity of care (BB index-score) practice-level (0-1)	0.416 (0.321-0.541)	0.397 (0.306-0.517)

[1] Diagnosed with one or more of the following seven chronic conditions: chronic renal disease, cancer, asthma, stroke, coronary heart disease, diabetes, or COPD. IMD=index of multiple deprivation, Q=quartiles, BB=Bice & Boxerman, IQ=inter-quartile range.

Table 2: Estimates of B-coefficients from multi-level regression (normal response) model for the association between introduction of named-GP and continuity of care (Bice & Boxerman index-score), England 2012-2016 (38,500 observations).

	**Unadjusted model**			**Adjusted model***		
	Coef.	p-value	95% CI	Coef.	p-value	95% CI
Constant	0.440	<0.001	0.413; 0.467	0.427	<0.001	0.404; 0.449
Period (ref.=pre)	-0.024	<0.001	-0.031; -0.018	-0.024	<0.001	-0.030; -0.017
Age (ref.= <75)	0.013	0.001	0.005; 0.021	0.017	<0.001	0.009; 0.025
Period * Age	-0.006	0.240	-0.015; 0.004	-0.005	0.342	-0.014; 0.005

*Co-variates set to average: gender, number of chronic conditions, level of deprivation (quintiles), number of GPs in practice (quintiles), number of GP consultations (quartiles), and urban/rural practice location. For complete table, see Supplementary Table 2.

For peer review only - http://bmjopen.bmj.com/site/about/guidelines.xhtml

Table 3: Estimates of odds ratios (OR) from multi-level regression (binomial logit) model for the association between introduction of named GP and risk of an emergency hospital admissions, England 2012-2016 (38,500 observations).

	**Unadjusted model**			**Adjusted model 1***			**Adjusted model 2****		
	OR	p-value	95% CI	OR	p-value	95% CI	OR	p-value	95% CI
Period (ref.=pre)	1.206	<0.001	1.111; 1.309	1.156	0.001	1.064; 1.257	1.137	0.007	1.035; 1.254
Age (ref.= <75)	1.887	<0.001	1.736; 2.048	1.594	<0.001	0.464; 1.735	1.680	<0.001	1.530; 1.846
Period * Age	1.179	0.003	1.059; 1.314	1.191	0.002	1.066; 1.330	1.228	0.001	1.086; 1.388

*Co-variates set to average: gender, number of chronic conditions, level of deprivation (quintiles), number of GPs in practice (quartiles), and urban/rural practice location. For complete table with B-coefficients, see Supplementary Table 3.
**Included also standardised covariates: number of GP consultations (quartiles) and continuity of care (Bice & Boxerman index-score, quartiles) till first emergency hospital admission or the end of the observation period when not admitted. Number of observations: 37,207.

Table 4: Estimates of rate ratios (RR) from multi-level regression (Poisson) model for the association between introduction of named GP and the number of emergency hospital admissions, England 2012-2016 (38,500 observations).

	**Unadjusted model**			**Adjusted model 1***			**Adjusted model 2****		
	RR	p-value	95% CI	RR	p-value	95% CI	RR	p-value	95% CI
Period (ref.=pre)	1.249	<0.001	1.170; 1.332	1.178	<0.001	1.103; 1.259	1.171	<0.001	1.097; 1.250
Age (ref.= 75-)	1.821	<0.001	1.687; 1,956	1.571	<0.001	1.462; 1.690	1.372	<0.001	1.280; 1.470
Period * Age	1.146	0.001	1.055; 1.245	1.143	0.001	1.052; 1.242	1.120	0.007	1.031; 1.215

*Co-variates set to average: gender, number of chronic conditions, level of deprivation (quintiles), number of GPs in practice (quartiles), and urban/rural practice location. For complete table, see Supplementary Table 4.
**Included also standardised covariates: number of GP consultations (quartiles) and continuity of care (Bice & Boxerman index-score, quartiles).

Figure legends

Figure 1: Distribution of Bice & Boxerman index-scores for April 2012-March 2014 (19,235 patients) and for April 2014-March 2016 (19,265 patients)

Figure 2: Estimates of B-coefficients from multi-level regression (normal response) model for the association between introduction of named-GP and continuity of care (Bice & Boxerman index-score), split according to level of practice-level continuity of care.

For peer review only - http://bmjopen.bmj.com/site/about/guidelines.xhtml

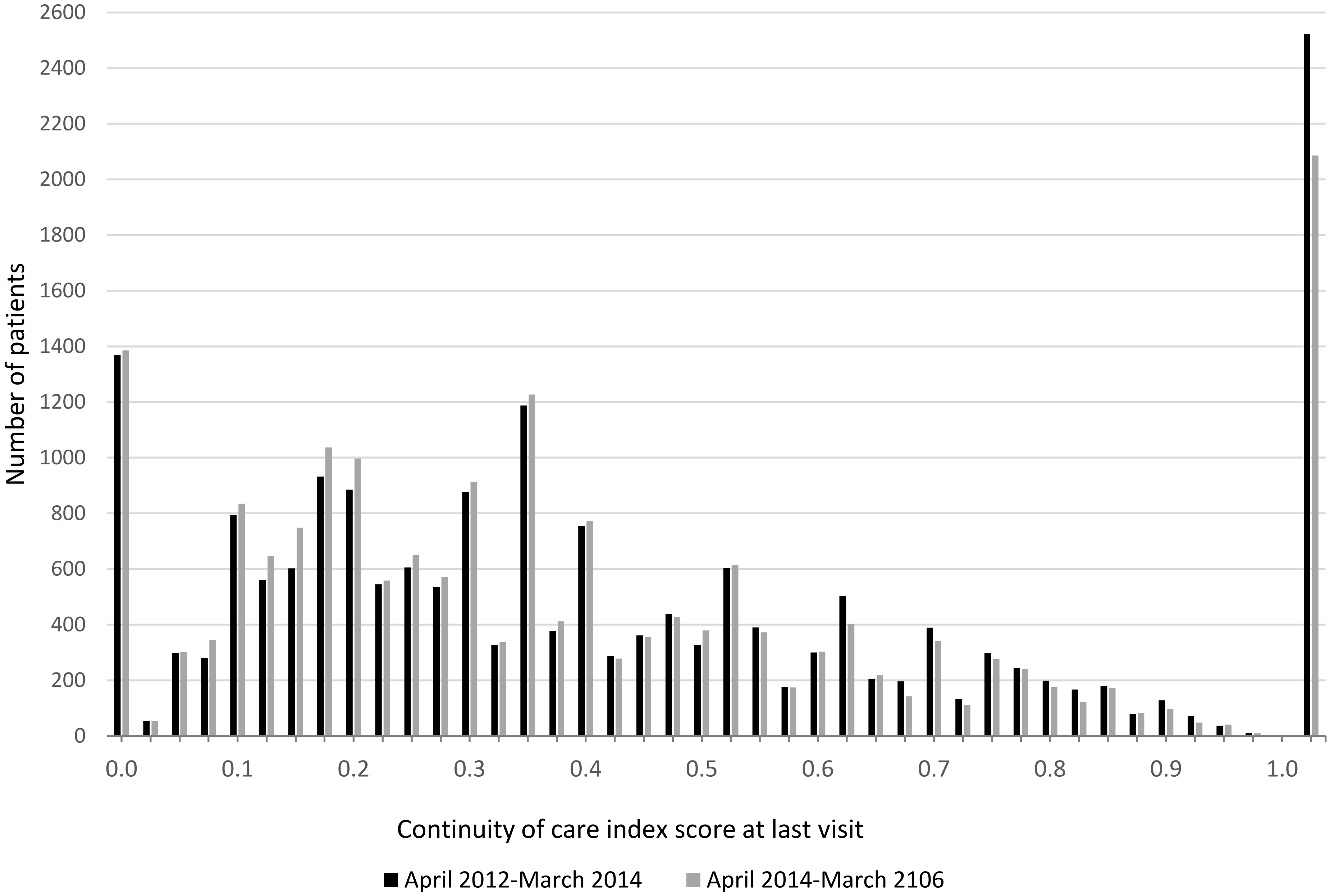
2600
2400
2200
2000
1800
1600
1400
1200
1000
800
600
400
200
0
Number of patients
0.0
0.1
0.2
0.3
0.4
0.5
0.6
0.7
0.8
0.9
1.0
Continuity of care index score at last visit
April 2012-March 2014
April 2014-March 2106

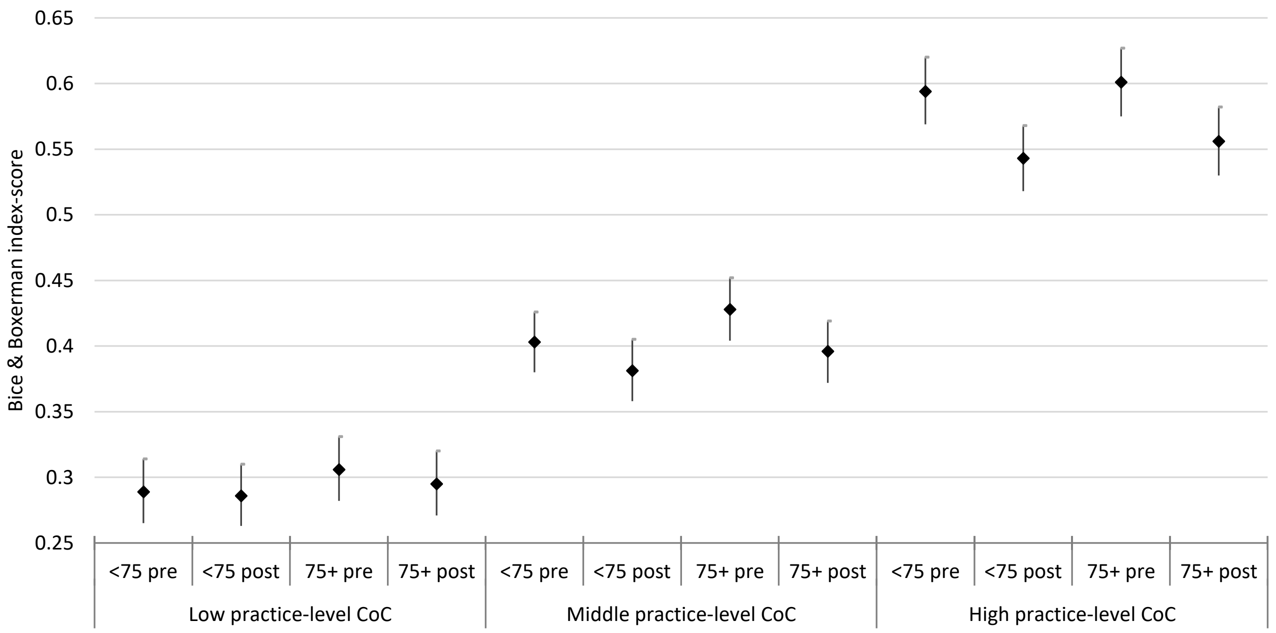
0.65
0.6
0.55
0.5
0.45
0.4
0.35
0.3
0.25
Bice & Boxerman index-score
<75 pre
<75 post
75+ pre
75+ post
Low practice-level CoC
Middle practice-level CoC
High practice-level CoC

Supplement

Supplementary Figure 1: Distribution of date when patients aged 75 and older were notified of named GP (n=12,526).

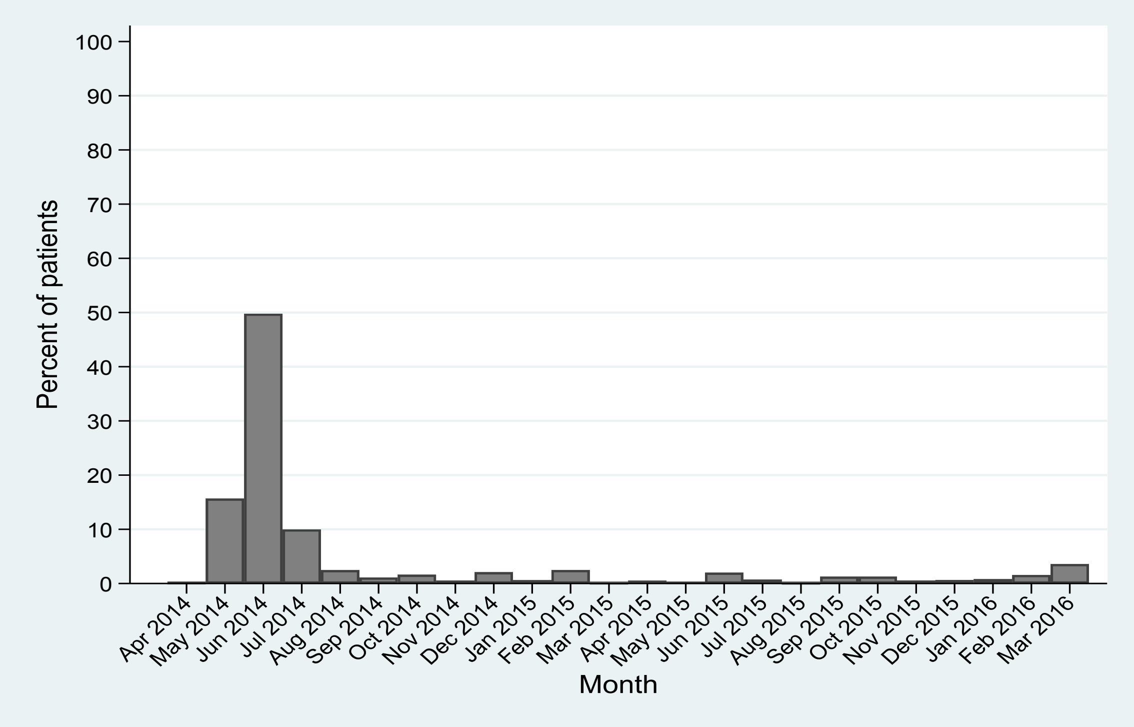

Supplementary Table 1: Cross-tabulation of continuity of care (using Bice & Boxerman index-scores) and number of GP consultations for a random sample of 38,500 patients in England between 2012 and 2016.

CoC (BB index)	**Number of GP consultations (%)**				
	2-5	6-9	10-15	16+	
Lowest CoC	3,941 (40.8)	1,946 (20.2)	1,869 (19.4)	1,896 (19.6)	9,652 (100.0)
CoC Qr2	1,454(15.6)	2,417 (25.9)	2,456 (26.3)	3,017 (32.3)	9,344 (100.0)
CoC Qr3	2,036 (20.7)	2,361 (24.0)	2,566 (26.0)	2,889 (29.3)	9,852 (100.0)
Highest CoC	3,032 (31.4)	2,441 (25.3)	2,172 (22.5)	2,007 (20.8)	9,652 (100.0)
Total	10,463 (27.2)	9,165 (23.8)	9,063 (23.5)	9,809 (25.5)	38,500 (100.0)

CoC=continuity of care, Qr=quartile, BB=Bice & Boxerman

For peer review only

Supplementary Table 2: Estimates of B-coefficients from multi-level regression (normal response) model for the association between introduction of named GP and continuity of care (using Bice & Boxerman index-scores) for a random sample of 38,500 patients in England between 2012 and 2016, adjusted for standardised covariates.

	Coef.	95% CI	p-value
Constant	0.427	0.404; 0.449	<0.001
Period (ref.=pre)	-0.024	-0.030; -0.017	<0.001
Age (ref.= 75-)	0.017	0.009; 0.025	<0.001
Period * Age	-0.005	-0.014; 0.005	0.344
Female (ref.=male)	-0.007	-0.010; -0.004	<0.001
Number of chronic morbidities[1]	-0.006	-0.009; -0.003	<0.001
Deprivation level Qn2 (ref.= Qn1-Least deprived)	0.001	-0.003; 0.005	0.503
Deprivation level Qn3 (ref.= Qn1-Least deprived)	-0.001	-0.006; 0.003	0.490
Deprivation level Qn4 (ref.= Qn1-Least deprived)	-0.003	-0.007; 0.001	0.142
Deprivation level Qn5 (ref.= Qn1-Least deprived)	-0.003	-0.007; 0.000	0.085
Number of GP consultations Qr2 (ref.=Qr1-lowest number)	0.002	-0.001; 0.005	0.255
Number of GP consultations Qr3 (ref.=Qr1-lowest number)	-0.001	-0.004; 0.003	0.716
Number of GP consultations Qr4 (ref.=Qr1-lowest number)	-0.004	-0.007; -0.000	0.051
Number of GPs in practice Qr2 (ref.=Qr1-lowest number)	-0.037	-0.046; -0.028	<0.001
Number of GPs in practice Qr3 (ref.=Qr1-lowest number)	-0.050	-0.061; -0.040	<0.001
Number of GPs in practice Qr4 (ref.=Qr1-lowest number)	-0.069	-0.081; -0.057	<0.001
Cities and towns (ref.=urban conurbation)	0.023	-0.009; 0.055	0.152
Rural (ref.=urban conurbation)	-0.017	-0.051; 0.018	0.342
Practice-level variance (constant)	0.017	0.013; 0.021	
Patient-level variance (constant)	0.017	0.016; 0.018	
Period-level variance (constant)	0.053	0.052; 0.054	

[1] Diagnosed with one or more of the following seven chronic conditions: chronic renal disease, cancer, asthma, stroke, coronary heart disease, diabetes, or COPD. Ref.= reference; Qn=quintile; Qr=quartile.

For peer review only

Supplementary Table 3: Estimates of B-coefficients from multi-level regression (binomial logit) model for association between introduction of named GP and risk of an emergency hospital admission for a random sample of 38,500 patients in England between 2012 and 2016, adjusted for standardised covariates.

	Coef.	95% CI	p-value
Constant	-2.019	-2.088; -1.951	<0.001
Period (ref.=pre)	0.145	0.062; 0.229	0.001
Age (ref.= 75-)	0.466	0.381; 0.551	<0.001
Period * Age	0.175	0.064; 0.285	0.002
Female (ref.=male)	-0.028	-0.057; 0.002	0.066
Number of chronic morbidities[1]	0.465	0.438; 0.492	<0.001
Deprivation level Qn2 (ref.= Qn1-Least deprived)	0.304	-0.007; 0.068	0.109
Deprivation level Qn3 (ref.= Qn1-Least deprived)	0.054	0.016; 0.92	0.005
Deprivation level Qn4 (ref.= Qn1-Least deprived)	0.065	0.029; 0.102	<0.001
Deprivation level Qn5 (ref.= Qn1-Least deprived)	0.083	0.049; 0.117	<0.001
Number of GPs in practice Qr2 (ref.=Qr1-lowest number)	-0.008	-0.056; 0.040	0.750
Number of GPs in practice Qr3 (ref.=Qr1-lowest number)	-0.004	-0.054; 0.047	0.887
Number of GPs in practice Qr4 (ref.=Qr1-lowest number)	0.001	-0.053; 0.055	0.974
Cities and towns (ref.=urban conurbation)	-0.061	-0.119; -0.003	0.039
Rural (ref.=urban conurbation)	-0.022	-0.083; 0.039	0.482
Practice-level variance (constant)	0.020	0.008; 0.033	
Patient-level variance (constant)	0.548	0.465; 0.632	

[1] Diagnosed with one or more of the following seven chronic conditions: chronic renal disease, cancer, asthma, stroke, coronary heart disease, diabetes, or COPD. Ref.= reference; Qn=quintile; Qr=quartile.

Note: the ratio of odds ratios for patients aged 75 and over was 1.19 compared with those under 75; this is calculated by taking the e raised power of 0.175.

Supplementary Table 4: Estimates of B-coefficients from multi-level regression (Poisson) model for association between introduction of named GP and the number of emergency hospital admissions for a random sample of 38,500 patients in England between 2012 and 2016, adjusted for standardised covariates.

	Coef.	95% CI	p-value
Constant	-1.868	-1.927; -1.808	<0.001
Period (ref.=pre)	0.164	0.098; 0.230	<0.001
Age (ref.= 75-)	0.452	0.380; 0.525	<0.001
Period * Age	0.134	0.051; 0.217	0.001
Female (ref.=male)	-0.024	-0.051; 0.003	0.087
Number of chronic morbidities[1]	0.428	0.403; 0.452	<0.001
Deprivation level Qn2 (ref.= Qn1-Least deprived)	0.036	0.002; 0.070	0.041
Deprivation level Qn3 (ref.= Qn1-Least deprived)	0.056	0.021; 0.91	0.002
Deprivation level Qn4 (ref.= Qn1-Least deprived)	0.070	0.037; 0.103	<0.001
Deprivation level Qn5 (ref.= Qn1-Least deprived)	0.086	0.055; 0.117	<0.001
Number of GPs in practice Qr2 (ref.=Qr1-lowest number)	0.004	-0.037; 0.045	0.845
Number of GPs in practice Qr3 (ref.=Qr1-lowest number)	0.014	-0.029; 0.058	0.525
Number of GPs in practice Qr4 (ref.=Qr1-lowest number)	0.004	-0.043; 0.050	0.887
Cities and towns (ref.=urban conurbation)	-0.058	-0.110; -0.006	0.028
Rural (ref.=urban conurbation)	-0.021	-0.076; 0.034	0.458
Practice-level variance (constant)	0.015	0.005; 0.025	
Patient-level variance (constant)	1.511	1.440; 1.582	

[1] Diagnosed with one or more of the following seven chronic conditions: chronic renal disease, cancer, asthma, stroke, coronary heart disease, diabetes, or COPD. Ref.= reference; Qn=quintile; Qr=quartile.

Note: the ratio of rate ratios for patients aged 75 and over was 1.14 compared with those under 75; this is calculated by taking the e raised power of 0.175.

STROBE Statement—Checklist of items that should be included in reports of ***cohort studies***

	Item No	Recommendation
**Title and abstract**	1	(*a*) Indicate the study's design with a commonly used term in the title or the abstract **[Within abstract's design section]**
		(*b*) Provide in the abstract an informative and balanced summary of what was done and what was found **[The abstract's setting, design and results sections]**
**Introduction**		
Background/rationale	2	Explain the scientific background and rationale for the investigation being reported **[Introduction on page 3]**
Objectives	3	State specific objectives, including any prespecified hypotheses **[Introduction on page 3]**
**Methods**		
Study design	4	Present key elements of study design early in the paper **[Abstract, and Methods page 4]**
Setting	5	Describe the setting, locations, and relevant dates, including periods of recruitment, exposure, follow-up, and data collection **[Abstract, and Methods page 4-6]**
Participants	6	(*a*) Give the eligibility criteria, and the sources and methods of selection of participants. Describe methods of follow-up **[Methods page 4]**
		(*b*) For matched studies, give matching criteria and number of exposed and unexposed **N/A**
Variables	7	Clearly define all outcomes, exposures, predictors, potential confounders, and effect modifiers. Give diagnostic criteria, if applicable **[Methods page 4-6]**
Data sources/ measurement	8*	For each variable of interest, give sources of data and details of methods of assessment (measurement). Describe comparability of assessment methods if there is more than one group **[Methods page 4]**
Bias	9	Describe any efforts to address potential sources of bias **[Strengths and weaknesses page 9]**
Study size	10	Explain how the study size was arrived at **[Methods page 4]**
Quantitative variables	11	Explain how quantitative variables were handled in the analyses. If applicable, describe which groupings were chosen and why **[Methods page 5-6]**
Statistical methods	12	(*a*) Describe all statistical methods, including those used to control for confounding **[Methods page 6, Results page 7-8, Discussion page 9]**
		(*b*) Describe any methods used to examine subgroups and interactions **[Methods page 6-7]**
		(*c*) Explain how missing data were addressed **[This study used anonymised data from patient's electronic records, we assume that all consultation data were recorded and all admissions to hospital.]**
		(*d*) If applicable, explain how loss to follow-up was addressed **[Methods page 4]**
		(*e*) Describe any sensitivity analyses
**Results**		
Participants	13*	(a) Report numbers of individuals at each stage of study—eg numbers potentially eligible, examined for eligibility, confirmed eligible, included in the study, completing follow-up, and analysed **[Methods page 4 and 6]**
		(b) Give reasons for non-participation at each stage **[Methods page 4]**
		(c) Consider use of a flow diagram **N/A**
Descriptive data	14*	(a) Give characteristics of study participants (eg demographic, clinical, social) and

For peer review only - http://bmjopen.bmj.com/site/about/guidelines.xhtml

		information on exposures and potential confounders **[Methods page 6, and table 1]**
		(b) Indicate number of participants with missing data for each variable of interest **N/A**
		(c) Summarise follow-up time (eg, average and total amount) **[Methods page 4]**
Outcome data	15*	Report numbers of outcome events or summary measures over time **[Methods page 5-6]**
Main results	16	(*a*) Give unadjusted estimates and, if applicable, confounder-adjusted estimates and their precision (eg, 95% confidence interval). Make clear which confounders were adjusted for and why they were included **[Methods page 6, Results 7-8]**
		(*b*) Report category boundaries when continuous variables were categorized **[Method page 7, Table 1]**
		(*c*) If relevant, consider translating estimates of relative risk into absolute risk for a meaningful time period **N/A**
Other analyses	17	Report other analyses done—eg analyses of subgroups and interactions, and sensitivity analyses **[Results page 7-8]**
**Discussion**		
Key results	18	Summarise key results with reference to study objectives **[Discussion page 9]**
Limitations	19	Discuss limitations of the study, taking into account sources of potential bias or imprecision. Discuss both direction and magnitude of any potential bias **[Discussion page 9-10]**
Interpretation	20	Give a cautious overall interpretation of results considering objectives, limitations, multiplicity of analyses, results from similar studies, and other relevant evidence **[Discussion page 10-11]**
Generalisability	21	Discuss the generalisability (external validity) of the study results **[Discussion page 11]**
**Other information**		
Funding	22	Give the source of funding and the role of the funders for the present study and, if applicable, for the original study on which the present article is based **[Page 12-13]**

*Give information separately for exposed and unexposed groups.

Note: An Explanation and Elaboration article discusses each checklist item and gives methodological background and published examples of transparent reporting. The STROBE checklist is best used in conjunction with this article (freely available on the Web sites of PLoS Medicine at http://www.plosmedicine.org/, Annals of Internal Medicine at http://www.annals.org/, and Epidemiology at http://www.epidem.com/). Information on the STROBE Initiative is available at http://www.strobe-statement.org.

For peer review only

BMJ Open

The impact of a named GP-scheme on continuity of care and emergency hospital admission. A cohort study among older patients in England, 2012-2016.

Journal:	*BMJ Open*
Manuscript ID	bmjopen-2019-029103.R1
Article Type:	Research
Date Submitted by the Author:	04-Jul-2019
Complete List of Authors:	Tammes, Peter; University of Bristol, Bristol Medical School<br>Payne, Rupert; University of Bristol, Centre for Academic Primary Care<br>Salisbury, Chris; University of Bristol, Centre for Academic Primary Care<br>Chalder, Melanie; University of Bristol, Centre for Academic Primary Care<br>Purdy, Sarah; University of Bristol, Centre for Academic Primary Care<br>Morris, Richard; University of Bristol, School of Social & Community Medicine
**Primary Subject Heading**:	Health services research
Secondary Subject Heading:	Health policy
Keywords:	Health policy < HEALTH SERVICES ADMINISTRATION & MANAGEMENT, Organisation of health services < HEALTH SERVICES ADMINISTRATION & MANAGEMENT, PRIMARY CARE, PUBLIC HEALTH, STATISTICS & RESEARCH METHODS

SCHOLARONE™
Manuscripts

The impact of a named GP-scheme on continuity of care and emergency hospital admission. A cohort study among older patients in England, 2012-2016.

Peter Tammes[1], Rupert Payne[1], Chris Salisbury[1], Melanie Chalder[1], Sarah Purdy[1] & Richard W Morris[1]

[1] University of Bristol, Bristol Medical School: Population Health Sciences, Centre for Academic Primary Care

Correspondence to Peter Tammes, Bristol Medical School: Population Health Sciences, University of Bristol, 39 Whatley Road, Bristol, BS8 2PS (e-mail: p.tammes@bristol.ac.uk) Phone: +44 (0) 117-928-7377; Fax: +44 (0) 117-928-7236

For peer review only

Key words: Health policy; organisation of health services; primary care; public health; statistics & research methods

Word count: 4073

Abstract

Objective:

To investigate whether the introduction of a named general practitioner (GP, family physician) improved patients' health care for patients aged 75 and over in England.

Setting

Random sample of 27,500 patients aged 65-84 in 2012 within 139 English practices from the Clinical Practice Research Datalink linked with Hospital Episode Statistics.

Design

Prospective cohort approach, measuring patients' GP consultations and emergency hospital admissions two years before/after the intervention. Patients were grouped in (i) aged over 74 and (ii) younger than 75 in both periods in order to compare who were or were not subject to the intervention. Adjusted associations between the named-GP scheme, continuity of care and emergency hospital admission were examined using multilevel modelling.

Intervention

National Health Service policy to introduce a named accountable GP for patients aged over 74 in April 2014.

Main outcome measures

A) continuity of care index-score, B) risk of emergency hospital admissions, C) number of emergency hospital admissions.

Results

The intervention was associated with a decrease in continuity index-scores of -0.024 (95% CI -0.030 to -0.017, p<0.001); there were no differences in the decrease between the two age groups (-0.005, 95% CI -0.014 to 0.005). In the pre- and post-intervention periods, respectively, 15.4% and 19.4% patients had an emergency admission. The probability of an emergency hospital admission increased after the intervention (OR 1.156, 95% CI 1.064 to 1.257, p=0.001); this increase was bigger for patients over 74 (relative OR 1.191, 95% CI 1.066 to 1.330, p=0.002). The average number of emergency hospital admissions increased after the intervention (RR 1.178, 95% CI 1.103 to 1.259, p<0.001); this increase was greater for patients over 74 (relative RR 1.143, 95% CI 1.052 to 1.242, p=0.001).

Conclusion

The introduction of the named-GP scheme was not associated with improvements in either continuity of care or rates of unplanned hospitalisation.

Strengths and limitations of this study

- This study was the first to investigate the relationship between the introduction of a named GP-scheme and continuity of primary care and unplanned hospitalisation.
- This study took a 4-year observational period into account, namely 2-year periods before and after the implementation of the named-GP policy.
- This cohort study used individual electronic healthcare records data from the Clinical Practice Research Datalink (CPRD) linked with Hospital Episode Statistics.
- Our dataset did not make it possible to specify the named GP assigned to a patient.
- As all older patients included in the sample survived the 4-year observational period, this might reduce the generalisability of our findings.

For peer review only - http://bmjopen.bmj.com/site/about/guidelines.xhtml

For peer review only

Introduction

Nearly every country in the world is experiencing growth in the number and proportion of older persons.[1] It is projected that by 2019 one in eleven people in the United Kingdom (UK) will be aged 75 or older, increasing to one in seven by 2040[2]. This demographic trend is likely to increase the number and proportion of people with long-term disability and chronic or multiple health conditions. To meet the challenges of an ageing population and to better serve those living with complex health and care needs, in April 2014 the National Health Service (NHS) Employers and General Medical Services (GMS) agreed to introduce a named accountable general practitioner (GP) [family or primary care physician] for all patients aged 75 or more. The aim was to provide personalised, proactive care to keep older people healthy, independent and out of hospital.[3 4]

In the UK, patients are registered at one general (family) practice but might see different physicians within that practice. The introduction of a named GP-scheme was intended to facilitate older patients consulting the same doctor, thus improving continuity of primary care (i.e. seeing the same clinician over time). As there is some evidence that continuity of primary care is declining in England[5], the named-GP scheme could potentially reverse this trend.

A key objective of the introduction of the named-GP scheme was to avoid or decrease hospital admission. Previous systematic reviews based on international literature concluded that better clinician continuity of care reduces hospitalisation.[6 7] There is some evidence from the UK that patients who do not see the same GP over a period of time are at higher risk of emergency hospital admission and have more admissions than those who see the same or a small number of GPs.[8 9] The aim of the current study was to investigate whether the named-GP scheme improved older patients' continuity of primary care, and decreased older patients' risk of emergency hospital admission and the number of such admissions. The findings of this study might also be relevant for other (Western) countries as they face the same burdens from an ageing population on their health care systems.

Methods

Study design and setting

This study used data from the Clinical Practice Research Datalink (CPRD) which contains anonymised electronic healthcare records on 4.4 million patients (6.9% of the UK population) and is nationally representative in terms of age, sex and ethnicity.[10] We obtained a random sample of 27,500 patients in 139 English GP-practices [family practices] who were aged 65 to 84 in 2012, alive in 2016, registered with their GP-practice for at least one year prior to 2012, and not transferred out of the

For peer review only - http://bmjopen.bmj.com/site/about/guidelines.xhtml

GP-practice during the study's observation period. The CPRD was deterministically linked with Hospital Episode Statistics (HES) to identify emergency hospital admissions in the financial years April 2012 – March 2016. This allowed us to compare a patient's individual healthcare use two years before the introduction of the named GP-scheme (April 2012-March 2014) with their healthcare use two years after its introduction (April 2014-March 2016). To measure the impact of the introduction of a named-GP scheme appropriately, we excluded patients who became 75 years old during the study (n=5,703) and created two age groups for patients who were aged 75 years and over in both periods (n=9,682), or aged under 75 years in both periods (n=12,115) in order to compare groups who were or were not subject to the intervention. Furthermore, analyses were restricted to only those patients with at least two GP consultations allowing to calculate continuity of primary care scores in either the pre- or post-intervention periods, totalling 19,235 patients in the pre-intervention period and 19,265 patients in the post-intervention period.

Based on our previous work, we expect 13.5% to undergo an emergency admission over a single year, therefore over 2 years, we estimate 22% will experience an admission.[9] The results of that study suggested that an upward shift across a quartile of the distribution of continuity of care might decrease risk by approximately 10%. Comparing a subsequent 2-year period in which the rate is reduced by 10% (i.e. rate ratio of 0.9) from 22% to 19.8% by introduction of the named-GP scheme for patients aged 75 and over, we have over 99% power to detect this difference at the 5% significance level at the given sample size for the pre- and post-intervention periods. If the rate is reduced by 6% (instead of 10%) to 20.7%, we have 88% power to detect this difference. These calculations were done in Stata 15.1 using sampsi and power commands.

Exposure

After the introduction of the named-GP scheme in April 2014, all patients aged 75 and over had to be notified by letter or during a consultation by 30 June 2014 of their named and accountable GP, or within 21 days after a patient became 75 or was newly registered if their practice was not already operating a personal list.[3] Practices were required to enter the Read Code "Informing patient of named accountable general practitioner" (code 67DJ) in the patient's health record. Based on the date of recording of this Read Code for all the 12,526 patients 75 and older in 2014 in the dataset, 65% were informed before 1 July 2014 and 75% before 1 August 2014. At the end of the observation period, i.e. 1 April 2016, 97% had been notified (see Supplementary Figure 1). We, however, did not exclude patients who were notified after 30 June 2014 for the purposes of our study as we did not know which practices already were operating a personal list. Furthermore, our analysis focused on the effects of the policy intervention as it was implemented; in effect, an intention-to-treat analysis.

For peer review only - http://bmjopen.bmj.com/site/about/guidelines.xhtml

Outcome measures

Three outcome measures were assessed, the first being change in continuity of primary care. We used a combination of CPRD staff codes to identify GP staff (senior partners, partners, salaried doctors, locum doctors, and GP registrars) within the practice and dates of consultations to identify whether these occurred during the pre-intervention or post-intervention period. Consultations included clinic and surgery consultations, home visits, out-of-hours' visits, telephone consultations, and third-party consultations. Following Hobbs et al. we did not restrict according to consultation duration.[11] Where a patient had more than one contact per day, we used information about staffing relating to the first consultation only, to avoid potential concerns about duplication of consultations. This information allows the quantification of continuity of care over 2-year periods pre-intervention and post-intervention. We calculated continuity of care using the Bice & Boxerman (BB) index[12], which ranges between 0 (complete absence of continuity) and 1 (perfect continuity of care), as this has been recommended for use in primary care research[13]. BB index-scores can be calculated for patients who consulted a GP more than once.

The second outcome assessed was change in probability of experiencing at least one emergency hospital admission after the second GP-consultation in both the pre- and post-intervention periods. We made no distinction between admissions by specific routes (e.g. patient self-presentation to the emergency department, GP referral to a hospital speciality). A patient's probability of at least one emergency hospital admission may not necessarily reflect the number of admissions a patient experienced. The probability might have decreased while the average number of emergency hospital admissions might have increased and vice versa. The third outcome, therefore, was the number of emergency hospital admissions after the second GP-consultation in both the pre- and post-intervention periods, categorised as zero, one, two, and three or more.

Covariates

Our choice of covariates was guided by the QAdmission score[14], previously developed using data from a similar routine general practitioner database to predict hospital admissions. Complete data were available on all patients in the sample with regard to age, gender, number of GP consultations, area-based socioeconomic Index of Multiple Deprivation 2015 in quintiles, location (conurbation, urban, rural), and the following morbidities measured between April 2010 and March 2014 for the pre-intervention period and measured between April 2012 and March 2016 for the post-intervention period. These included diagnoses made in the 2 years prior to the pre- and post-intervention periods, using published clinical code lists as collected in the Manchester Clinical Codes repository[15]: epilepsy[16], chronic kidney disease[17], cancer[18], asthma[17], stroke[19], coronary heart

For peer review only - http://bmjopen.bmj.com/site/about/guidelines.xhtml

disease[19], diabetes[19], COPD[16], depression[20], and schizophrenia[20]. Furthermore, we took into account clustering at the practice level as practice factors might facilitate or reduce continuity of care[21] and estimated the number of GPs in a practice using patient's GP consultations and staff role information for each general practice. Descriptive statistics are provided in Table 1.

To adjust for continuity of care (BB index-score) for the second outcome, we determined the continuity of care until an emergency hospital admission or to the end of each period (whichever came first) when not having experienced such an emergency admission, excluding from the analysis patients who experienced an emergency admission before their first or second GP consultation (as these patients would not have a continuity score). This resulted in a reduction in the of number of observations from 38,500 to 37,207. The BB index-scores were divided into quintiles.

Table 1: Descriptive statistics

	Pre-intervention (19,235)	Post-intervention (19,265)
	N (Pct.)	N (Pct.)
Patients younger than 75	10,404 (54.1)	10,368 (53.8)
Patients 75 or older	8,831 {45.9)	8,897 (46.2)
Male	8,699 (45.2)	8,698 (45.2)
Female	10.536 (54.8)	10,567 (54.8)
Least deprived, quintile 1	5,294 (27.5)	5,340 (27.7)
Deprivation quintile 2	4,395 (22.9)	4,421 (23.0)
Deprivation quintile 3	4,266 (22.6)	4,238 (22.0)
Deprivation quintile 4	3,195 (16.6)	3,194 (16.6)
Most deprived, quintile 5	2,084 (10.8)	2,071 (10.7)
2-5 GP consultations (2-5), quintile 1	5,333 (27.7)	5,130 (26.6)
6-9 GP consultations, quintile 2	4,697 (24.4)	4,468 (23.2)
10-15 GP consultations, quintile 3	4,545 (23.6)	4,518 (23.5)
16 or more GP consultations (16+), quintile 4	4,660 (24.2)	5,149 (26.7)
Less than 9 GPs in practice pre-intervention (post: <8), quintile 1	2,977 (15.5)	2,764 (14.4)
9-14 GPs in practice pre-intervention (post: 8-13), quintile 2	4,715 (24.5)	4,555 (23.6)
15-21 GPs in practice pre-intervention (post: 14-22), quintile 3	5,032 (26.2)	5,996 (31.1)
More than 21 GPs in practice pre-intervention (post >22), quintile 4	6,511 (33.9)	5,950 (30.9)
Urban conurbation	6,145 (32.0)	6,180 (32.1)
Cities and towns	10,207 (53.1)	10,290 (53.4)
Rural	2,883 (15.0)	2,795 (14.5)
No emergency hospital admission	16,269 (84.6)	15,520 (80.6))
1 emergency hospital admission	2,070 (10.8)	2,368 (12.3)
2 emergency hospital admissions	557 (2.9)	750 (3.9)
More than 2 emergency hospital admissions	339 (1.7)	627 (3.2)
	Median (IQ)	Median (IQ)
Total number of morbidities[1] (0-6)	1 (0-1)	1 (0-1)
Continuity of care (BB index-score) patient-level (0-1)	0.344 (0.184-0.622)	0.333 (0.167-0.574)
Continuity of care (BB index-score) practice-level (0-1)	0.416 (0.321-0.541)	0.397 (0.306-0.517)

[1] Diagnosed with one or more of the following seven chronic conditions: chronic renal disease, cancer, asthma, stroke, coronary heart disease, diabetes, or COPD. IMD=index of multiple deprivation, Q=quartiles, BB=Bice & Boxerman, IQ=inter-quartile range.

Statistical methods

A patient's BB index and a patient's emergency hospital admission were measured for both the pre-intervention and the post-intervention periods. To account for repeated measurements by time, by patient and by practice, this study used multilevel modelling. Because continuity of care was a

For peer review only - http://bmjopen.bmj.com/site/about/guidelines.xhtml

continuous variable, a normal response regression model was used to associate the named-GP scheme with continuity of care (BB index-score). Because experiencing at least one emergency hospital admission was a binary variable, a binomial logit regression model to associate the named-GP scheme with risk of emergency hospital admissions was used. A Poisson regression model was used to associate the named-GP scheme with the number of emergency hospital admissions as this outcome was a count.

To represent whether the effect of the intervention operated differently for patients aged over 75 (exposed) from those aged under 75 (unexposed), we included the age × period interaction. This could be interpreted as difference in change of the BB index score, the relative odds ratio of emergency hospital admission or the relative rate ratio of number of emergency hospital admissions. We used Stata 15.1 to perform our analyses.

Results

Outcome: continuity of primary care

The distribution of the BB index varied widely, with similarity between the pre and post-intervention distributions (Figure 1). Respectively, 1,365 (7.1%) and 2,523 (13.1%) patients never or always saw the same GP in the pre-intervention period: equivalent numbers were 1,376 (7.1%) and 2,086 (10.8%) in the post-intervention period. The change in BB index-score over time varied also widely (see Supplementary Figure 2) with an interquartile range between -0.190 and 0.141.

Figure 1 here

The BB index-score decreased over time by 0.028 (from 0.428 in the pre-intervention period to 0.399 in the post-intervention); this equates to a drop in the mean continuity of care by about 6.5%. The BB index-score for patients aged 75 and over decreased from 0.434 pre-intervention to 0.403 post-intervention (a mean decrease of 0.031). This decrease was slightly bigger than for patients younger than 75, from 0.422 pre-intervention to 0.397 post-intervention (a drop of 0.025). An unadjusted multilevel (normal response) model for continuity of care (BB index-score) showed there was no evidence that this decrease in continuity of care following the intervention differed between the two age groups (Table 2, time-age interaction -0.006 (95% CI -0.015; 0.004)). As patients in the lowest or highest continuity of care quartiles consulted a GP less often than those in the middle two quartiles (see Supplementary Table 1), we included number of consultations in the analysis as one of the co-

For peer review only - http://bmjopen.bmj.com/site/about/guidelines.xhtml

variates together with other factors such as gender, number of chronic conditions, socioeconomic deprivation, number of GPs in practice, and rurality (see Supplementary Table 2). This adjusted model still showed no significant difference in the decrease of continuity of care over time between the two age groups (Table 2, time-age interaction -0.005 (95%CI -0.014; 0.005)). Continuity of care declined in both the unexposed and exposed groups and there was no evidence of the decline being stronger in one of the groups.

Table 2: Estimates of B-coefficients from multi-level regression (normal response) model for the association between introduction of named-GP and continuity of care (Bice & Boxerman index-score), England 2012-2016 (38,500 observations).

	**Unadjusted model**			**Adjusted model***		
	Coef.	p-value	95% CI	Coef.	p-value	95% CI
Constant	0.440	<0.001	0.413; 0.467	0.427	<0.001	0.404; 0.449
Period (ref.=pre)	-0.024	<0.001	-0.031; -0.018	-0.024	<0.001	-0.030; -0.017
Age (ref.= <75)	0.013	0.001	0.005; 0.021	0.017	<0.001	0.009; 0.025
Period * Age	-0.006	0.240	-0.015; 0.004	-0.005	0.342	-0.014; 0.005

*Co-variates set to average: gender, number of chronic conditions, level of deprivation (quintiles), number of GPs in practice (quintiles), number of GP consultations (quartiles), and urban/rural practice location. For complete table, see Supplementary Table 2.

Sensitivity analysis

We also calculated for each practice the average practice-level continuity of care score over 2012-2014, having divided practices into tertiles: low, middle, high continuity of care. This allowed us to determine whether patients in practices with different levels of continuity of care show differing trends in continuity of care post-intervention. The result of an interaction between period, age, and practice-level continuity of care is illustrated in Figure 2. The continuity of care of patients in a practice with generally low continuity of care dropped less between pre- and post-introduction of the named-GP scheme for both patients younger and older than 75 compared to practices with generally middle and high practice-level continuity of care.

Figure 2 here

<u>Outcome: risk of emergency hospital admission</u>

In the pre-intervention and the post-intervention periods, respectively, 2,966 (15.4%) and 3,745 (19.4%) patients had one or more emergency admissions. The probability of an emergency hospital admission for patients aged 75 and over showed an absolute increase of 6.3%-points over time (from 19.9% pre-intervention to 26.2% post-intervention). There was evidence from the unadjusted

For peer review only - http://bmjopen.bmj.com/site/about/guidelines.xhtml

model that the relative increase in odds of admission was 17.9% (95%CI 5.9%-31.4%) greater in those aged over 75 years than those aged under 75 years after the introduction of the named-GP scheme (Table 3). This relative difference between age groups persisted (19.1%, 95%CI 6.6%-33.0%) after adjustment for other factors such as gender, number of chronic conditions, socioeconomic deprivation, number of GPs in practice, and rurality (see Supplementary Table 3). The relative difference between age groups was marginally greater following additional adjustment for continuity of care (BB index-score estimated until the event date) and number of GP consultations (22.8%, 95%CI 8.6%-38.8%).

Table 3: Estimates of odds ratios (OR) from multi-level regression (binomial logit) model for the association between introduction of named GP and risk of an emergency hospital admissions, England 2012-2016 (38,500 observations).

	**Unadjusted model**			**Adjusted model 1***			**Adjusted model 2****		
	OR	p-value	95% CI	OR	p-value	95% CI	OR	p-value	95% CI
Period (ref.=pre)	1.206	<0.001	1.111; 1.309	1.156	0.001	1.064; 1.257	1.137	0.007	1.035; 1.254
Age (ref.= <75)	1.887	<0.001	1.736; 2.048	1.594	<0.001	0.464; 1.735	1.680	<0.001	1.530; 1.846
Period * Age	1.179	0.003	1.059; 1.314	1.191	0.002	1.066; 1.330	1.228	0.001	1.086; 1.388

*Co-variates set to average: gender, number of chronic conditions, level of deprivation (quintiles), number of GPs in practice (quartiles), and urban/rural practice location. For complete table with B-coefficients, see Supplementary Table 3.
**Included also standardised covariates: number of GP consultations (quartiles) and continuity of care (Bice & Boxerman index-score, quartiles) till first emergency hospital admission or the end of the observation period when not admitted. Number of observations: 37,207.

<u>Outcome: number of emergency hospital admissions</u>

In the pre-intervention period 16,269 (84.6%), 2,070 (10.7%), 557 (2.9%), and 339 (1.76%) experienced respectively no, one, two, or three or more emergency hospital admissions. In the post-intervention period 15,520 (80.6%), 2,368 (12.3%), 750 (3.9%), and 627 (3.3%) experienced respectively no, one, two, or three or more emergency hospital admissions. The mean number of emergency hospital admissions for patients aged 75 and over showed an absolute increase of 0.154 over time (from 0.313 pre-intervention to 0.467 post-intervention). There was evidence from the unadjusted model that the relative increase in mean number of emergency hospital admissions after the introduction of the named-GP scheme was 14.6% (95%CI 5.5%-24.5%) greater in those aged over 75 years than those aged under 75 years (Table 4, Unadjusted model). This relative difference between age groups persisted (14.3%, 95%CI 5.2%-24.2%) after adjustment for other factors such as gender, number of chronic conditions, socioeconomic deprivation, number of GPs in practice, and rurality (Table 4, Adjusted model 1; see Supplementary Table 4).

For peer review only - http://bmjopen.bmj.com/site/about/guidelines.xhtml

Sensitivity analysis

To adjust for continuity of care (BB index-score) and for number of GP consultations we determined the continuity of care at the end of each period for all patients included in the analysis, in contrast to the other outcome where continuity of care was estimated only until the event date. In this model the relative difference between the two age groups was slightly lower than in the unadjusted model (12%, 95%CI 3.1-21.5%) (Table 4, Adjusted model 2).

Table 4: Estimates of rate ratios (RR) from multi-level regression (Poisson) model for the association between introduction of named GP and the number of emergency hospital admissions, England 2012-2016 (38,500 observations).

	**Unadjusted model**			**Adjusted model 1***			**Adjusted model 2****		
	RR	p-value	95% CI	RR	p-value	95% CI	RR	p-value	95% CI
Period (ref.=pre)	1.249	<0.001	1.170; 1.332	1.178	<0.001	1.103; 1.259	1.171	<0.001	1.097; 1.250
Age (ref.= 75-)	1.821	<0.001	1.687; 1,956	1.571	<0.001	1.462; 1.690	1.372	<0.001	1.280; 1.470
Period * Age	1.146	0.001	1.055; 1.245	1.143	0.001	1.052; 1.242	1.120	0.007	1.031; 1.215

*Co-variates set to average: gender, number of chronic conditions, level of deprivation (quintiles), number of GPs in practice (quartiles), and urban/rural practice location. For complete table, see Supplementary Table 4.
**Included also standardised covariates: number of GP consultations (quartiles) and continuity of care (Bice & Boxerman index-score, quartiles).

Discussion

Principal findings

Continuity of care decreased between the pre- and post-intervention periods and this decrease was similar for patients aged between 65 and 74 (who were not eligible for the named-GP scheme over the period of study) and patients aged 75 and over (who were eligible). Over time, continuity of care for patients aged 75 years or over declined less in practices which had lowest continuity of care at baseline. The average decrease in continuity of care was small, about 6.5% from baseline, although there was considerable variation across patients and practices. The probability of an emergency hospital admission increased between the pre- and post-intervention periods and this increase was greater for patients 75 and over. The average number of emergency hospital admissions also increased, and this increase was greater for patients aged 75 and over. In general, the introduction of a named-GP scheme was not associated with improvements in either continuity of care or rates of emergency hospital admissions.

Emergency hospital admission showed a stronger increase among patients aged 75 and over, contrary to what we expected, but we don't think that this is associated to the measured decrease in

For peer review only - http://bmjopen.bmj.com/site/about/guidelines.xhtml

continuity of care as patients 75 and over and those younger than 75 experienced a similar drop in continuity. It is unclear whether the increase is due to the named-GP system mediated through some other mechanism than continuity of care, or whether it's due to other factors not captured by our study.

Strengths and weaknesses of the study

This study used longitudinal individual-level data from older patients in the CPRD to assess continuity of care and its relationship with the incidence of unplanned hospital admission before and after the introduction of a named GP-scheme, by comparing patients assigned a named GP with slightly younger patients not assigned a named GP. This allowed us to determine and compare continuity of care and unplanned hospital admission over time and between the affected and unaffected group. The observation period was a 2-year period before and after the introduction of a named GP-scheme, allowing us to calculate robust continuity of care scores for each period using the BB index-scores. The dataset allowed us also to control for practice-level, clinical and demographic co-variates.

The study had some limitations. Firstly, all the patients included in the sample survived the 4-year observational period. This may indicate that we had a particularly 'healthy' group of older patients and might, therefore, reduce the generalisability of the study's findings. Our dataset did not make it possible to specify the named GP assigned to a patient, therefore we could not use other measures of continuity of care such as the provider identification index.[22]

Comparison with other studies

Lloyd and Steventon published a protocol for a regression discontinuity study to investigate the effect of the introduction of the named GP-scheme on the number of GP contacts per patients, the number of GP referrals to specialists, and the number of common diagnostic tests.[23] Following up on their protocol, Barker, Lloyd and Steventon did not find any associations with their outcomes of interest measured over 9 months following assignment to a named accountable GP and attributed this to their short period under study.[24] The present study took a longer observational period into account, namely 2-year periods before and after the implementation, resulting in more robust findings. Whereas study of Barker, Lloyd and Steventon focused on number of GP contacts, our study explored the possible effect on continuity of care, since one of the mechanisms by which assigning a named GP to a patient could have an impact might be by increasing continuity. Furthermore, Lloyd and Steventon's outcome measures reflect only primary care service use. As a key objective of the introduction of a named GP-scheme was to avoid or decrease hospital

For peer review only

admission, this study also calculated associations between the introduction of a named GP and risk and number of emergency hospital admissions.

Using aggregated practice-level data from the GP-patient survey, Levene et al. showed that the proportion of patients seeing their preferred GP dropped between 2012 and 2017, especially in practices with higher percentages of those aged 75 and older.[25] Based on this result they questioned the effectiveness of the named GP-policy for older patients. Our study showed a decrease as well in continuity of care, measured by BB index-scores. However, as our study used individual-data, avoiding the ecological fallacy, we showed that the decrease in continuity of care was similar for those aged between 65-74 and for those aged 75 and older. Possibly because most patients were already listed at a GP list and introducing a named-GP policy for older patients might not have changed much to their situation of being allocated to a GP. We were also able to determine that continuity of care of patients in a practice providing on average low continuity of care dropped less compared to patients in practices providing on average high continuity of care, which may be an example of regression to the mean.

Meaning of the study: possible explanations and implications for clinicians and policymakers

The named GP-scheme for older patients was introduced by the NHS, with each individual general practice having to assign doctors to older patients on their list. The General Medical Services Contract did not advise practices to consult patients about their preferred GP as part of this assignment process, nor did it guarantee that patients would see the same clinician at each consultation. However, where patients expressed a preference as to which GP they have been assigned, the practice had to make reasonable effort to accommodate these requests.[3] In most general practices in the UK patients were already nominally allocated to a particular GP within a practice on the practice computer system, because until 2004 patients were registered with an individual GP rather than a practice. However, patients may not have been aware of this, and the GP named on the computer system may have had little or no significance for patient care.[26] The main change introduced with the named GP policy was informing patients of the GP who was accountable for their care. This did not necessarily reflect which GP the patient had seen most often or take into account whether the patient had a preferred GP. Even though the impact might therefore have been expected to be small, this study still provides insights into whether or not this policy has impacted on continuity of care, as well as whether it has achieved its aims of reducing hospitalisation.

Allocating a GP does not imply that patients are able to see or speak to that GP whenever they require advice or care since this depends on GP workload, practice opening hours, salaried and part-time working contracts.[27] The importance of continuity of care in the patient-doctor relationship is

For peer review only - http://bmjopen.bmj.com/site/about/guidelines.xhtml

much more complex than the simple allocation of a named doctor. Other factors that may be important, particularly in the context of reducing future emergency admissions, are the education of patients over a period of time, and knowledge of a patient's usual health status.[28] These are reflections of the depth of the relationship between the patient and doctor – which will not automatically be improved by the allocation of a named doctor to a particular patient.[29]

A policy of allocating a named GP in itself is not effective and more sophisticated interventions would be needed to improve continuity of care in the UK or countries with similar healthcare systems. However, it is not possible to tell from our study whether applying an assigned named-GP scheme in a country where continuity of care is not common, might actually lead to improvements in continuity of care and, consequently, hospital use.

Unanswered questions and future research

Future research might focus on differences between practices concerning the implementation of the named GP scheme. As this research showed a difference between patients in general practices with on average low versus high continuity of care, a number of other differences could impact implementation, such as practice size and proportion of part-time GPs. Our study focused on continuity of care and unplanned hospital admission, future research using a 2-year or even longer observational period might focus on other healthcare use such as number of GP referrals or diagnostic tests[24], drug prescription and medication adherence. A complication, however, might be the introduction of a named-GP scheme for all patients in April 2015 which should have been implement in all practices before April 2016.[30] Though, the named GP for patients younger than 75 has largely a role of oversight for a patient's health in contrast to the named GP for patients 75 and over who should actively provide personalised care.

This study does not investigate the views and experiences of patients or practice staff. Evidence suggests that older patients value continuity of care[27], but qualitative research or surveys could explore whether they identified any change in care after the introduction of the named GP scheme. We also do not know whether the scheme led to any meaningful changes in how practices offered care to patients and or in the extent to which individual GPs felt accountable for particular patients. Qualitative research in practices could usefully explore this issue in order to improve implementation of a named GP-scheme.

For peer review only - http://bmjopen.bmj.com/site/about/guidelines.xhtml

Data Availability

Our data were obtained through licensing agreements (Protocol 17_140R) with the Clinical Practice Research Datalink (CPRD). The CPRD is the new English National Health Service (NHS) observational data and interventional research service, jointly funded by the NHS National Institute for Health Research (NIHR) and the Medicines and Healthcare products Regulatory Agency (MHRA). All access and use of data via the CPRD are carefully controlled under UK and European law and the rules and regulations operating in the NHS. In accordance with the conditions of the CPRD license, data abstracts from the CPRD could not be deposited in a public data depository. Data are available to other researchers upon request from the CPRD (http://www.cprd.com/contact/), and approval by the CPRD Independent Scientific Committee (ISAC, http://www.cprd.com/ISAC/).

Competing interests

None declared

Authors' contribution

PT and RWM designed the study. PT managed and performed the analysis. CS, MC, RP, and SP contributed to the methodological approach and also added significant input to the results and discussion. All authors contributed to the interpretation of the findings. PT was the lead investigator for the overall project.

Patient and Public Involvement in Research

At the start of the study, PT presented an outline of the project to the Elizabeth Blackwell Institute (EBI) Public Advisory Group based at the University of Bristol. Feedback from this meeting was used by MC to plan a range of engagement and involvement activities with older people and, where appropriate, their carers and family members. All activities took place in community settings, focusing on the concept of the policy initiative, the planned research design and the study results. Information from both the EBI Public Advisory Group and the discussions with patients and members of the public guided the preparation of data for analysis and informed the interpretation of the results. A plain English summary of the study findings has been prepared for dissemination alongside the scientific and academic outputs. A series of appropriately targeted publications for lay audiences has also been planned.

Data sharing

For peer review only - http://bmjopen.bmj.com/site/about/guidelines.xhtml

The data controller of the data analysed is the Clinical Practice Research Datalink. Patient-level data is available subject to their information governance requirements.

Ethical approval

This study was approved by the CPRD Independent Scientific Advisory Committee (ISAC) committee. Protocol no.: 17_140R.

Transparency declaration

The lead author affirms that this manuscript is an honest, accurate, and transparent account of the study being reported; that no important aspects of the study have been omitted; and that any discrepancies from the study as planned (and, if relevant, registered) have been explained.

Funding Statement

This work was funded by the National Institute for Health Research School of Primary Care Research (NIHR SPCR) grant funded round 13, PI PT project number 337. The views expressed are those of the authors and not necessarily those of the NIHR, NHS or Department of Health.

This study is based in part on data from the Clinical Practice Research Datalink obtained under licence from the UK Medicines and Healthcare products Regulatory Agency. The data is provided by patients and collected by the NHS as part of their care and support. The interpretation and conclusions contained in this study are those of the author/s alone.

Copyright © 2017, re-used with the permission of The Health & Social Care Information Centre. All rights reserved.

References

1. United Nations, Department of Economic and Social Affairs, Population Division. World Population Ageing 2017 (ST/ESA/SER.A/408). New York, 2017.
2. Government Office for Science. Future of an Ageing Population. Foresight Report. London: Government Office for Science, 2016.
3. General Medical Services Contract 2014/15 Guidance and Audit Requirements. NHS England Gateway reference: British Medical Association, 2014.
4. Lea W. Transforming primary care. Safe, proactive, personalised care for those who need it most. London: Department of Health, 2014.
5. Baker M, Jeffers H. Continuity of care in modern day general practice. London: royal College of General Practitioners, 2016.

For peer review only - http://bmjopen.bmj.com/site/about/guidelines.xhtml

6. Van Walraven C, Oake N, Jennings A, et al. The association between continuity of care and outcomes: a systematic and critical review. *Journal of evaluation in clinical practice* 2010;16(5):947-56.
7. Huntley A, Lasserson D, Wye L, et al. Which features of primary care affect unscheduled secondary care use? A systematic review. *BMJ open* 2014;4(5):e004746.
8. Barker I, Steventon A, Deeny S. Continuity of care in general practice is associated with fewer hospital admissions for ambulatory care sensitive conditions: a cross-sectional study of routinely collected, person-level data. *BMJ* 2017
9. Tammes P, Purdy S, Salisbury C, et al. Continuity of primary care and emergency hospital admissions among older patients in England. *The Annals of Family Medicine* 2017;15(6):515-22.
10. Herrett E, Gallagher AM, Bhaskaran K, et al. Data Resource Profile: Clinical Practice Research Datalink (CPRD). *International journal of epidemiology* 2015;44(3):827-36.
11. Hobbs FR, Bankhead C, Mukhtar T, et al. Clinical workload in UK primary care: a retrospective analysis of 100 million consultations in England, 2007–14. *The Lancet* 2016;387(10035):2323-30.
12. Bice TW, Boxerman SB. A quantitative measure of continuity of care. *Medical care* 1977;15(4):347-49.
13. Salisbury C, Sampson F, Ridd M, et al. How should continuity of care in primary health care be assessed? *British Journal of General Practice* 2009;59(561):e134-e41.
14. Hippisley-Cox J, Coupland C. Predicting risk of emergency admission to hospital using primary care data: derivation and validation of QAdmissions score. *BMJ open* 2013;3(8):e003482.
15. Springate DA, Kontopantelis E, Ashcroft DM, et al. ClinicalCodes: an online clinical codes repository to improve the validity and reproducibility of research using electronic medical records. *PloS one* 2014;9(6):e99825.
16. Reilly S, Olier I, Planner C, et al. Inequalities in physical comorbidity: a longitudinal comparative cohort study of people with severe mental illness in the UK. *BMJ open* 2015;5(12):e009010.
17. Doran T, Kontopantelis E, Valderas JM, et al. Effect of financial incentives on incentivised and non-incentivised clinical activities: longitudinal analysis of data from the UK Quality and Outcomes Framework. *Bmj* 2011;342:d3590.
18. Reeves D, Springate DA, Ashcroft DM, et al. Can analyses of electronic patient records be independently and externally validated? The effect of statins on the mortality of patients with ischaemic heart disease: a cohort study with nested case–control analysis. *BMJ open* 2014;4(4):e004952.
19. Kontopantelis E, Springate D, Reeves D, et al. Withdrawing performance indicators: retrospective analysis of general practice performance under UK Quality and Outcomes Framework. *Bmj* 2014;348:g330.
20. Carr MJ, Ashcroft DM, Kontopantelis E, et al. Clinical management following self-harm in a UK-wide primary care cohort. *Journal of affective disorders* 2016;197:182-88.
21. Kristjansson E, Hogg W, Dahrouge S, et al. Predictors of relational continuity in primary care: patient, provider and practice factors. *BMC family practice* 2013;14(1):72.
22. Starfield BH, Simborg DW, Horn SD, et al. Continuity and coordination in primary care: their achievement and utility. *Medical care* 1976;14(7):625-36.
23. Lloyd T, Steventon A. Effect of Named, Accountable GPs on Continuity of Care: Protocol for a Regression Discontinuity Study of a National Policy Change. *International Journal of Integrated Care* 2016;16(1)
24. Barker I, Lloyd T, Steventon A. Effect of a national requirement to introduce named accountable general practitioners for patients aged 75 or older in England: regression discontinuity analysis of general practice utilisation and continuity of care. *BMJ Open* 2016;6(9):e011422.
25. Levene LS, Baker R, Walker N, et al. Predicting declines in perceived relationship continuity using practice deprivation scores: a longitudinal study in primary care. 2018:bjgp18X696209.

For peer review only - http://bmjopen.bmj.com/site/about/guidelines.xhtml

26. Goodwin N, Dixon A, Poole T, et al. Improving the quality of care in general practice: Report of an independent inquiry commissioned by The King's Fund. 2011
27. Palmer W, Hemmings N, Rosen R, et al. Improving access and continuity in general practice: practical and policy lessons. Research summary. London: Nuffield Trust, 2018.
28. Simmonds RL, Shaw A, Purdy S. Factors influencing professional decision making on unplanned hospital admission: a qualitative study. *Br J Gen Pract* 2012;62(604):e750-e56.
29. Merriel SWD, Salisbury C, Metcalfe C, et al. Depth of the patient–doctor relationship and content of general practice consultations: cross-sectional study. *Br J Gen Pract* 2015;65(637):e545-e51.
30. British Medical Association, NHS Employers, NHS England. Technical requirements for 2015/16 gms contract changes, 2015.

Figure legends

Figure 1: Distribution of Bice & Boxerman index-scores for April 2012-March 2014 (19,235 patients) and for April 2014-March 2016 (19,265 patients)

Figure 2: Estimates of B-coefficients (95% CIs) from multi-level regression (normal response) model for the association between introduction of named-GP and continuity of care (Bice & Boxerman index-score), split according to level of practice-level continuity of care.

For peer review only

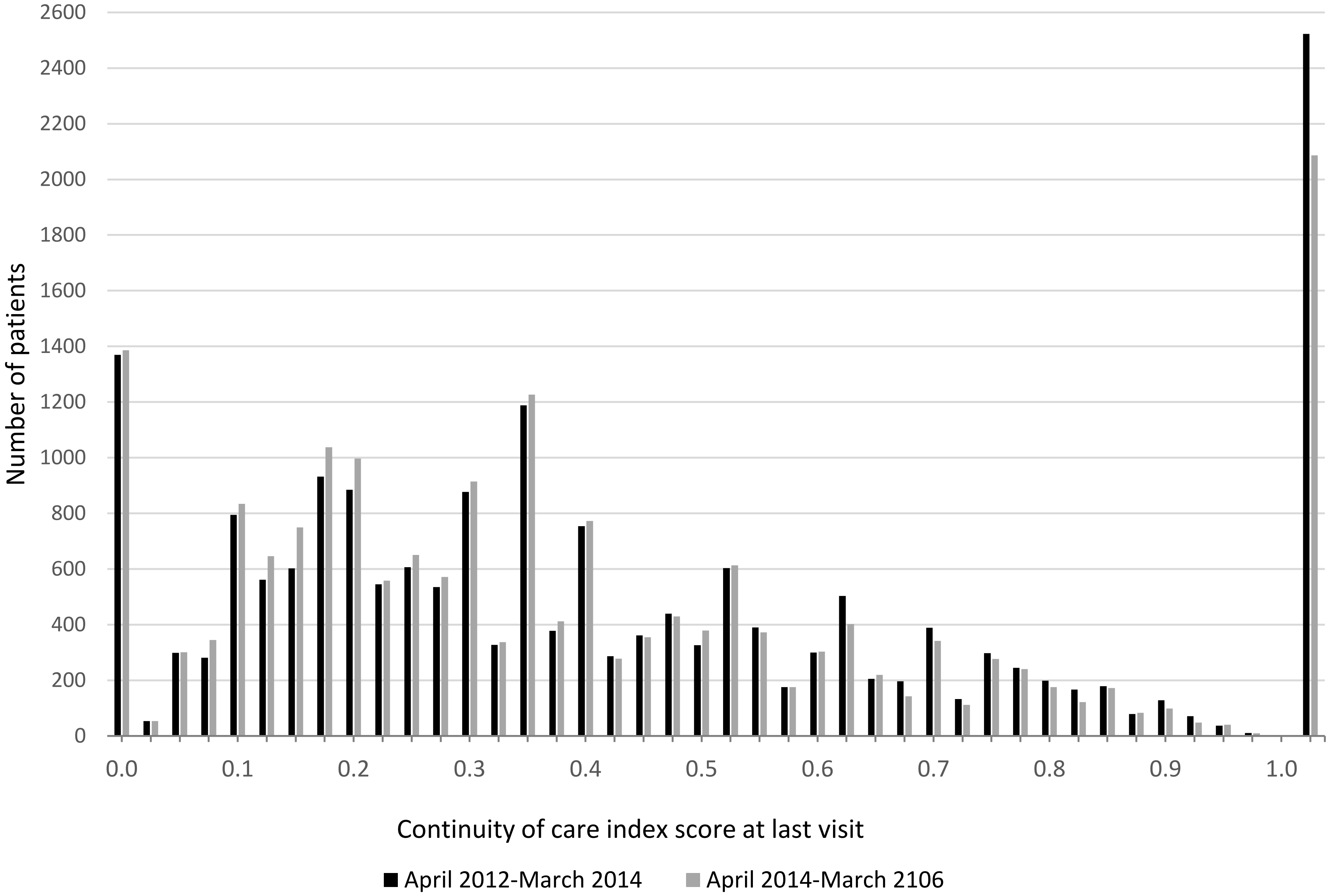
Number of patients
2600
2400
2200
2000
1800
1600
1400
1200
1000
800
600
400
200
0
0.0
0.1
0.2
0.3
0.4
0.5
0.6
0.7
0.8
0.9
1.0
Continuity of care index score at last visit
April 2012-March 2014
April 2014-March 2106

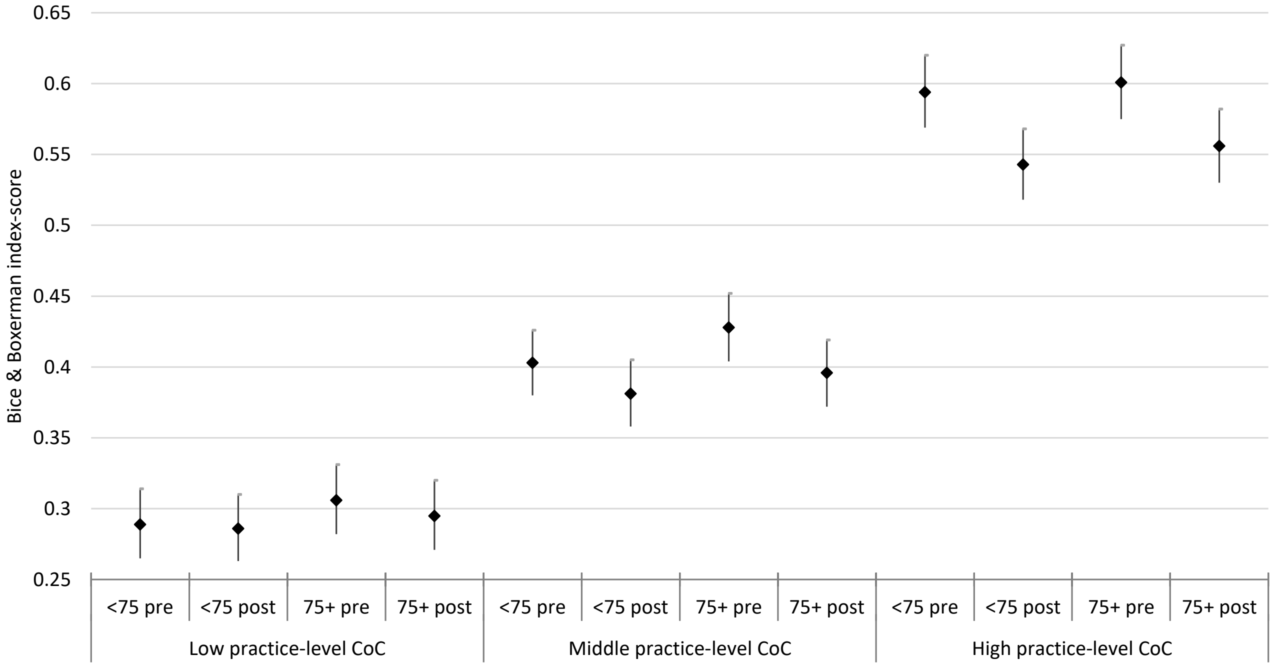
0.65
0.6
0.55
0.5
0.45
0.4
0.35
0.3
0.25
Bice & Boxerman index-score
<75 pre
<75 post
75+ pre
75+ post
Low practice-level CoC
Middle practice-level CoC
High practice-level CoC

Supplement

Supplementary Figure 1: Distribution of date when patients aged 75 and older were notified of named GP (n=12,526).

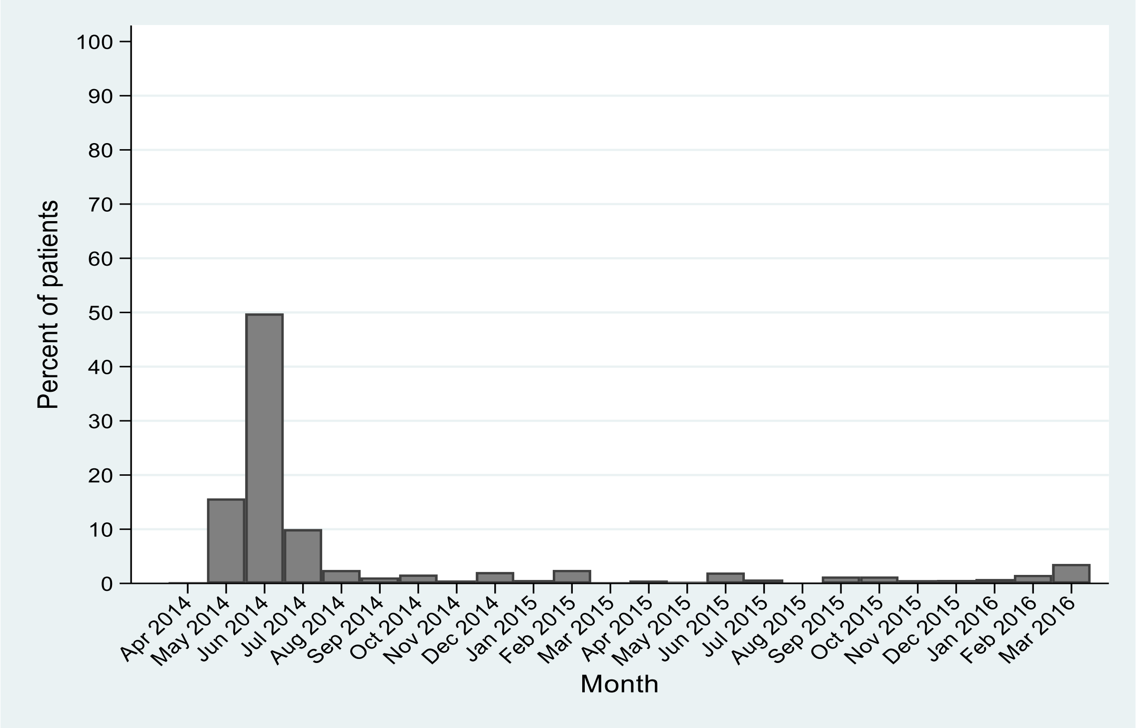

Supplementary Figure 2: Distribution of change in BB index-score between 2012-14 and 2014-16 for patients aged 65-70 (N=9,502) and 75 or older (N=8,409).

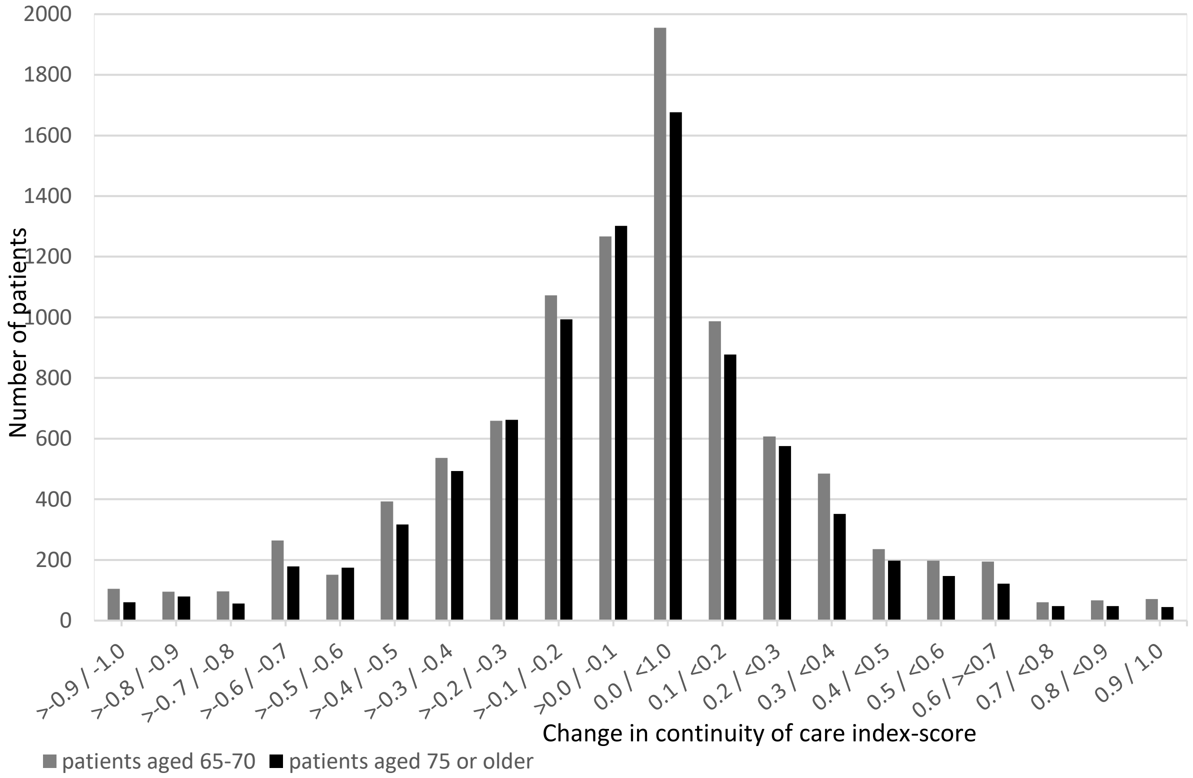

Supplementary Table 1: Cross-tabulation of continuity of care (using Bice & Boxerman index-scores) and number of GP consultations for a random sample of 38,500 patients in England between 2012 and 2016.

CoC (BB index)	**Number of GP consultations (%)**				
	2-5	6-9	10-15	16+	
Lowest CoC	3,941 (40.8)	1,946 (20.2)	1,869 (19.4)	1,896 (19.6)	9,652 (100.0)
CoC Qr2	1,454(15.6)	2,417 (25.9)	2,456 (26.3)	3,017 (32.3)	9,344 (100.0)
CoC Qr3	2,036 (20.7)	2,361 (24.0)	2,566 (26.0)	2,889 (29.3)	9,852 (100.0)
Highest CoC	3,032 (31.4)	2,441 (25.3)	2,172 (22.5)	2,007 (20.8)	9,652 (100.0)
Total	10,463 (27.2)	9,165 (23.8)	9,063 (23.5)	9,809 (25.5)	38,500 (100.0)

CoC=continuity of care, Qr=quartile, BB=Bice & Boxerman

Supplementary Table 2: Estimates of B-coefficients from multi-level regression (normal response) model for the association between introduction of named GP and continuity of care (using Bice & Boxerman index-scores) for a random sample of 38,500 patients in England between 2012 and 2016, adjusted for standardised covariates.

	Coef.	95% CI	p-value
Constant	0.427	0.404; 0.449	<0.001
Period (ref.=pre)	-0.024	-0.030; -0.017	<0.001
Age (ref.= 75-)	0.017	0.009; 0.025	<0.001
Period * Age	-0.005	-0.014; 0.005	0.344
Female (ref.=male)	-0.007	-0.010; -0.004	<0.001
Number of chronic morbidities[1]	-0.006	-0.009; -0.003	<0.001
Deprivation level Qn2 (ref.= Qn1-Least deprived)	0.001	-0.003; 0.005	0.503
Deprivation level Qn3 (ref.= Qn1-Least deprived)	-0.001	-0.006; 0.003	0.490
Deprivation level Qn4 (ref.= Qn1-Least deprived)	-0.003	-0.007; 0.001	0.142
Deprivation level Qn5 (ref.= Qn1-Least deprived)	-0.003	-0.007; 0.000	0.085
Number of GP consultations Qr2 (ref.=Qr1-lowest number)	0.002	-0.001; 0.005	0.255
Number of GP consultations Qr3 (ref.=Qr1-lowest number)	-0.001	-0.004; 0.003	0.716
Number of GP consultations Qr4 (ref.=Qr1-lowest number)	-0.004	-0.007; -0.000	0.051
Number of GPs in practice Qr2 (ref.=Qr1-lowest number)	-0.037	-0.046; -0.028	<0.001
Number of GPs in practice Qr3 (ref.=Qr1-lowest number)	-0.050	-0.061; -0.040	<0.001
Number of GPs in practice Qr4 (ref.=Qr1-lowest number)	-0.069	-0.081; -0.057	<0.001
Cities and towns (ref.=urban conurbation)	0.023	-0.009; 0.055	0.152
Rural (ref.=urban conurbation)	-0.017	-0.051; 0.018	0.342
Practice-level variance (constant)	0.017	0.013; 0.021	
Patient-level variance (constant)	0.017	0.016; 0.018	
Period-level variance (constant)	0.053	0.052; 0.054	
intraclass correlation coefficient (ICC)	0.195		

[1] Diagnosed with one or more of the following seven chronic conditions: chronic renal disease, cancer, asthma, stroke, coronary heart disease, diabetes, or COPD. Ref.= reference; Qn=quintile; Qr=quartile.

For peer review only

Supplementary Table 3: Estimates of B-coefficients from multi-level regression (binomial logit) model for association between introduction of named GP and risk of an emergency hospital admission for a random sample of 38,500 patients in England between 2012 and 2016, adjusted for standardised covariates.

	Coef.	95% CI	p-value
Constant	-2.019	-2.088; -1.951	<0.001
Period (ref.=pre)	0.145	0.062; 0.229	0.001
Age (ref.= 75-)	0.466	0.381; 0.551	<0.001
Period * Age	0.175	0.064; 0.285	0.002
Female (ref.=male)	-0.028	-0.057; 0.002	0.066
Number of chronic morbidities[1]	0.465	0.438; 0.492	<0.001
Deprivation level Qn2 (ref.= Qn1-Least deprived)	0.304	-0.007; 0.068	0.109
Deprivation level Qn3 (ref.= Qn1-Least deprived)	0.054	0.016; 0.92	0.005
Deprivation level Qn4 (ref.= Qn1-Least deprived)	0.065	0.029; 0.102	<0.001
Deprivation level Qn5 (ref.= Qn1-Least deprived)	0.083	0.049; 0.117	<0.001
Number of GPs in practice Qr2 (ref.=Qr1-lowest number)	-0.008	-0.056; 0.040	0.750
Number of GPs in practice Qr3 (ref.=Qr1-lowest number)	-0.004	-0.054; 0.047	0.887
Number of GPs in practice Qr4 (ref.=Qr1-lowest number)	0.001	-0.053; 0.055	0.974
Cities and towns (ref.=urban conurbation)	-0.061	-0.119; -0.003	0.039
Rural (ref.=urban conurbation)	-0.022	-0.083; 0.039	0.482
Practice-level variance (constant)	0.020	0.008; 0.033	
Patient-level variance (constant)	0.548	0.465; 0.632	
Intraclass correlation coefficient (ICC)	0.035		

[1] Diagnosed with one or more of the following seven chronic conditions: chronic renal disease, cancer, asthma, stroke, coronary heart disease, diabetes, or COPD. Ref.= reference; Qn=quintile; Qr=quartile.

Note: the ratio of odds ratios for patients aged 75 and over was 1.19 compared with those under 75; this is calculated by taking the e raised power of 0.175.

For peer review only

Supplementary Table 4: Estimates of B-coefficients from multi-level regression (Poisson) model for association between introduction of named GP and the number of emergency hospital admissions for a random sample of 38,500 patients in England between 2012 and 2016, adjusted for standardised covariates.

	Coef.	95% CI	p-value
Constant	-1.868	-1.927; -1.808	<0.001
Period (ref.=pre)	0.164	0.098; 0.230	<0.001
Age (ref.= 75-)	0.452	0.380; 0.525	<0.001
Period * Age	0.134	0.051; 0.217	0.001
Female (ref.=male)	-0.024	-0.051; 0.003	0.087
Number of chronic morbidities[1]	0.428	0.403; 0.452	<0.001
Deprivation level Qn2 (ref.= Qn1-Least deprived)	0.036	0.002; 0.070	0.041
Deprivation level Qn3 (ref.= Qn1-Least deprived)	0.056	0.021; 0.91	0.002
Deprivation level Qn4 (ref.= Qn1-Least deprived)	0.070	0.037; 0.103	<0.001
Deprivation level Qn5 (ref.= Qn1-Least deprived)	0.086	0.055; 0.117	<0.001
Number of GPs in practice Qr2 (ref.=Qr1-lowest number)	0.004	-0.037; 0.045	0.845
Number of GPs in practice Qr3 (ref.=Qr1-lowest number)	0.014	-0.029; 0.058	0.525
Number of GPs in practice Qr4 (ref.=Qr1-lowest number)	0.004	-0.043; 0.050	0.887
Cities and towns (ref.=urban conurbation)	-0.058	-0.110; -0.006	0.028
Rural (ref.=urban conurbation)	-0.021	-0.076; 0.034	0.458
Practice-level variance (constant)	0.015	0.005; 0.025	
Patient-level variance (constant)	1.511	1.440; 1.582	
Intraclass correlation coefficient (ICC)	0.010		

[1] Diagnosed with one or more of the following seven chronic conditions: chronic renal disease, cancer, asthma, stroke, coronary heart disease, diabetes, or COPD. Ref.= reference; Qn=quintile; Qr=quartile.

Note: the ratio of rate ratios for patients aged 75 and over was 1.14 compared with those under 75; this is calculated by taking the e raised power of 0.134.

For peer review only

STROBE Statement—Checklist of items that should be included in reports of ***cohort studies***

	Item No	Recommendation
**Title and abstract**	1	(*a*) Indicate the study's design with a commonly used term in the title or the abstract **[Within abstract's design section]**
		(*b*) Provide in the abstract an informative and balanced summary of what was done and what was found **[The abstract's setting, design and results sections]**
**Introduction**		
Background/rationale	2	Explain the scientific background and rationale for the investigation being reported **[Introduction on page 3]**
Objectives	3	State specific objectives, including any prespecified hypotheses **[Introduction on page 3]**
**Methods**		
Study design	4	Present key elements of study design early in the paper **[Abstract, and Methods page 4]**
Setting	5	Describe the setting, locations, and relevant dates, including periods of recruitment, exposure, follow-up, and data collection **[Abstract, and Methods page 4-6]**
Participants	6	(*a*) Give the eligibility criteria, and the sources and methods of selection of participants. Describe methods of follow-up **[Methods page 4]**
		(*b*) For matched studies, give matching criteria and number of exposed and unexposed **N/A**
Variables	7	Clearly define all outcomes, exposures, predictors, potential confounders, and effect modifiers. Give diagnostic criteria, if applicable **[Methods page 4-6]**
Data sources/ measurement	8*	For each variable of interest, give sources of data and details of methods of assessment (measurement). Describe comparability of assessment methods if there is more than one group **[Methods page 4]**
Bias	9	Describe any efforts to address potential sources of bias **[Strengths and weaknesses page 9]**
Study size	10	Explain how the study size was arrived at **[Methods page 4]**
Quantitative variables	11	Explain how quantitative variables were handled in the analyses. If applicable, describe which groupings were chosen and why **[Methods page 5-6]**
Statistical methods	12	(*a*) Describe all statistical methods, including those used to control for confounding **[Methods page 6, Results page 7-8, Discussion page 9]**
		(*b*) Describe any methods used to examine subgroups and interactions **[Methods page 6-7]**
		(*c*) Explain how missing data were addressed **[This study used anonymised data from patient's electronic records, we assume that all consultation data were recorded and all admissions to hospital.]**
		(*d*) If applicable, explain how loss to follow-up was addressed **[Methods page 4]**
		(*e*) Describe any sensitivity analyses
**Results**		
Participants	13*	(a) Report numbers of individuals at each stage of study—eg numbers potentially eligible, examined for eligibility, confirmed eligible, included in the study, completing follow-up, and analysed **[Methods page 4 and 6]**
		(b) Give reasons for non-participation at each stage **[Methods page 4]**
		(c) Consider use of a flow diagram **N/A**
Descriptive data	14*	(a) Give characteristics of study participants (eg demographic, clinical, social) and

For peer review only - http://bmjopen.bmj.com/site/about/guidelines.xhtml

		information on exposures and potential confounders **[Methods page 6, and table 1]**
		(b) Indicate number of participants with missing data for each variable of interest **N/A**
		(c) Summarise follow-up time (eg, average and total amount) **[Methods page 4]**
Outcome data	15*	Report numbers of outcome events or summary measures over time **[Methods page 5-6]**
Main results	16	(*a*) Give unadjusted estimates and, if applicable, confounder-adjusted estimates and their precision (eg, 95% confidence interval). Make clear which confounders were adjusted for and why they were included **[Methods page 6, Results 7-8]**
		(*b*) Report category boundaries when continuous variables were categorized **[Method page 7, Table 1]**
		(*c*) If relevant, consider translating estimates of relative risk into absolute risk for a meaningful time period **N/A**
Other analyses	17	Report other analyses done—eg analyses of subgroups and interactions, and sensitivity analyses **[Results page 7-8]**
**Discussion**		
Key results	18	Summarise key results with reference to study objectives **[Discussion page 9]**
Limitations	19	Discuss limitations of the study, taking into account sources of potential bias or imprecision. Discuss both direction and magnitude of any potential bias **[Discussion page 9-10]**
Interpretation	20	Give a cautious overall interpretation of results considering objectives, limitations, multiplicity of analyses, results from similar studies, and other relevant evidence **[Discussion page 10-11]**
Generalisability	21	Discuss the generalisability (external validity) of the study results **[Discussion page 11]**
**Other information**		
Funding	22	Give the source of funding and the role of the funders for the present study and, if applicable, for the original study on which the present article is based **[Page 12-13]**

*Give information separately for exposed and unexposed groups.

Note: An Explanation and Elaboration article discusses each checklist item and gives methodological background and published examples of transparent reporting. The STROBE checklist is best used in conjunction with this article (freely available on the Web sites of PLoS Medicine at http://www.plosmedicine.org/, Annals of Internal Medicine at http://www.annals.org/, and Epidemiology at http://www.epidem.com/). Information on the STROBE Initiative is available at http://www.strobe-statement.org.

For peer review only
